# Pathogenicity, transmissibility, and receptor binding of a human-isolated influenza A (H10N5) virus

**DOI:** 10.1128/mbio.00731-25

**Published:** 2025-07-08

**Authors:** Mengchan Hao, Jiaying Wu, Lina Ji, Yubo Zhao, Shunyuan Zhang, Yiwei Guan, Liangyu Li, Wenxue Yang, Yuan Zhang, Jianjun Chen

**Affiliations:** 1 State Key Laboratory of Virology and Biosafety, Wuhan Institute of Virology, Chinese Academy of Sciences 74614 , Wuhan, People’s Republic of China; 2 University of Chinese Academy of Sciences 74519 , Beijing, People’s Republic of China; 3 School of Life Sciences, Inner Mongolia University 12576 , Hohhot, People’s Republic of China; 4 Department of Pulmonary and Critical Care Medicine, Renmin Hospital of Wuhan University 117921, Wuhan, People’s Republic of China; St. Jude Children's Research Hospital, Memphis, Tennessee, USA

**Keywords:** novel H10N5, replication, pathogenicity, transmissibility, receptor binding

## Abstract

Recently, human infections with H10 influenza viruses, including H10N8 and H10N3, have been reported. In January 2024, a case of H10N5 and H3N2 co-infection was reported in Zhejiang Province, China, which is the first human infection with H10N5 avian influenza virus (AIV) globally. Almost simultaneously, we isolated a wild bird-derived H10N5 strain similar to the human H10N5 strain. To assess the public health risk, it is necessary to understand the zoonotic characteristics of these novel H10N5 viruses. Here, we evaluated the biological characteristics of human H10N5, wild bird H10N5, as well as poultry H10N8 *in vitro* and *in vivo*. We demonstrate that the novel H10N5 isolates infected and replicated effectively in human lung epithelial cells. They infected BALB/c mice without adaptation, which exhibited robust pathogenicity and caused mouse death. In guinea pig transmission experiments, the H10N5 strain spread through neither direct contact nor airborne exposure, whereas H10N8 transmitted effectively. Additionally, H10N5 exhibited dual receptor-binding characteristics with a stronger preference for avian receptors. The current public health risk of H10N5 is low. However, the occasional spillover infections of H10 AIV into humans and dual receptor-binding characteristics suggest a potential risk of cross-species transmission.

**IMPORTANCE:**

In 2024, the H10N5 AIV was first reported to infect humans. Concurrently, we isolated a strain of H10N5 from wild birds that was highly similar to the human H10N5 strain. However, the zoonotic potential and the associated public health risks of the H10N5 virus remain unclear. In this study, we systematically evaluated the replication characteristics of human H10N5, wild bird H10N5, and poultry H10N8 in human lung epithelial cells, the virulence in mice, the transmission capabilities in guinea pigs, and the receptor-binding properties. Our results demonstrate that these novel H10N5 viruses have not yet acquired the ability to transmit in guinea pigs, but they possess the potential to infect mammals. These findings provide timely insights and warnings for the development of public health prevention strategies.

## INTRODUCTION

The avian influenza virus (AIV) is a significant zoonotic virus that causes acute respiratory infections in a broad host range, including poultry, wild birds, mammals, and even humans. Generally, the natural host of AIV is considered to be wild waterfowl. Recently, AIV has frequently spread from birds to infect humans with novel zoonotic strains, including H3N8, H5N1, H7N9, and H10N8, emerging as ongoing public health threats (1–4).

The H10 subtype AIV was first isolated from chickens in Germany in 1949 (5), and it is predominantly found in poultry and wild bird populations across China (6). However, occasional cross-species transmission from birds to mammals has also been observed. H10 AIV has occasionally spilled over into humans. To date, cases of human infection with various H10 subtypes (H10N3, H10N7, and H10N8) have been reported (7–9). In January 2024, the World Health Organization confirmed the first human case of H10N5 AIV co-infection with H3N2 seasonal influenza virus. The co-infection caused severe pneumonia and type I respiratory failure, ultimately leading to death (10). Epidemiological investigations indicate that the patient had a history of exposure to live poultry infected with H10N5 (11). Almost concurrently, we isolated a wild bird-derived H10N5 strain (A/wild birds/Hubei/YYH40/2024, WB/2024) that is highly similar to human H10N5 with a common ancestor in multiple gene segments (12).

Before H10N5 virus infection, humans have been infected with H10N8 and H10N3 viruses. Studies show that almost all human H10 isolates are closely related to avian H10 viruses, with wild bird strains contributing to their generation (13, 14). Biological characteristics show that H10N3 is highly pathogenic in mice and can be transmitted between guinea pigs via respiratory droplets (15). H10N8 can bind human-like receptors and transmit efficiently among chickens and ducks (16). Human-to-human transmission has not been reported. However, the potential risk of continued cross-species transmission of H10 AIV from poultry and wild birds to humans deserves high attention.

To better understand the zoonotic characteristics and potential public health threats posed by the novel H10N5 virus, we systematically evaluated their biological properties. We investigated the infectivity and pathogenicity of H10N5 strains isolated from humans and wild birds alongside H10N8 strains isolated from poultry in 2013 using human lung epithelial cells and mammalian models. Moreover, we characterized the host receptor-binding properties and assessed its transmissibility in the guinea pig model.

## RESULTS

### Phylogenetic analysis of H10 viruses and representative strain selection

**Fig 1:**
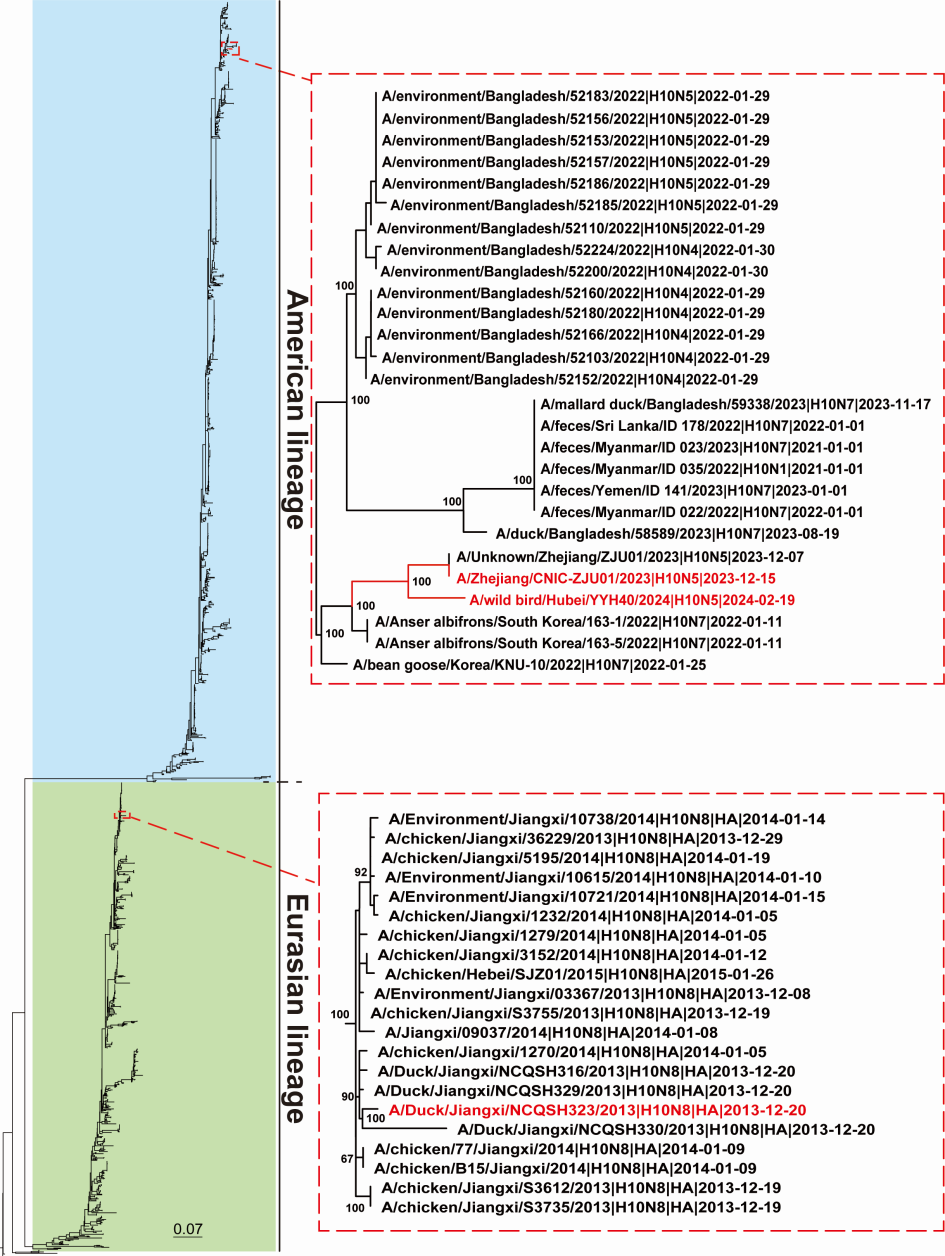
Phylogenetic analysis. The maximum likelihood phylogenetic tree of the hemagglutinin (HA) gene with coding sequences for H10 influenza virus. A total of 1,889 sequences of the HA gene were obtained from the GISAID influenza virus public database. Tree stability was determined by bootstrap analysis with 1,000 replicates and best-fit nucleotide substitution models using IQ-TREE2.

We conducted a phylogenetic analysis of the first human H10N5 strain, including our recently isolated H10 strain and other H10 strains from the GISAID influenza virus database. The results showed that the hemagglutinin (HA) phylogenetic tree was divided into the North American and Eurasian lineages. The wild bird-derived H10 A/wild birds/Hubei/YYH40/2024, WB/2024 (H10N5), which was isolated in our lab, had the closest genetic relationship with the first human-derived H10 A/Zhejiang/CNIC-ZJU01/2023, ZJ/2023 (H10N5) (Table S1). These two strains belonged to the North American lineage (Fig. 1). Human isolate ZJ/2023 (H10N5) and wild bird strain WB/2024 (H10N5) were selected for subsequent experiments. Humans infected with poultry-derived H10N8 were reported in 2013 (4). We subsequently isolated the H10N8 strain from ducks in a poultry market, which showed high genomic similarity to that of the human H10N8 isolate (data not shown). Thus, duck-derived H10N8 A/Duck/Jiangxi/NCQSH323/2013, DK/2013 (H10N8), which belonged to the Eurasian lineage, was included in subsequent experiments.

### Novel H10N5 replicates efficiently in human lung epithelial cells

**Fig 2:**
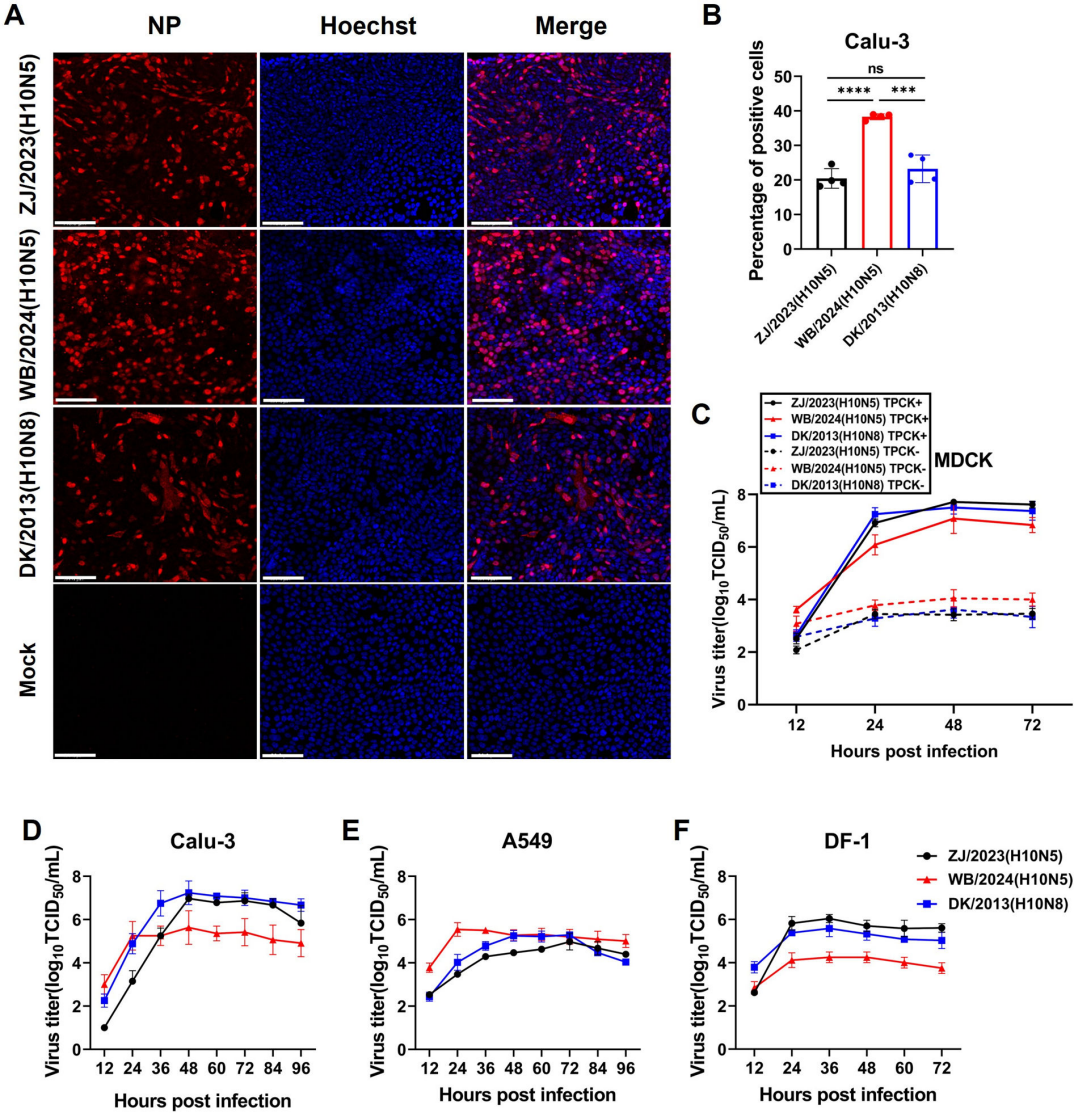
Infection and growth kinetics of novel H10N5 viruses in cells. (**A**) Calu-3 cells were infected with the indicated virus at an MOI of 1.0 for a single-cycle infectivity assay. At 8 h post-infection (hpi), infected cells were fixed with 4% paraformaldehyde and stained using an anti-influenza virus nucleoprotein (NP) antibody to detect the expression levels of the influenza NP protein. Nuclei were stained with Hoechst 33258. Merged images from both channels are shown in the right column. Scale bar: 100 µm. (**B**) The percentage of influenza virus NP protein-positive cells was quantified in infected cells. The values are expressed as the mean percentage ± standard deviation from three randomly selected fields. (**C**) The viral growth characteristics of the novel H10N5 virus in the MDCK cell with or without TPCK-treated trypsin were evaluated. Supernatants were collected at 12, 24, 48, and 72 hpi, respectively, and the viral titers were titrated on MDCK. The values are expressed as the mean ± standard deviation (*n* = 3). (**D–F**) Calu-3, A549, and DF-1 were grown on a 24-well culture plate and infected with the indicated virus at an MOI of 0.01. Cells were incubated at 37°C with 5% CO_2_. Culture supernatants were harvested at 12, 24, 36, 48, 60, 72, 84, and 96 hpi, respectively, and titrated in MDCK. The data shown consist of three independent replicates and are presented as mean ± standard deviation. ***P < 0.001, ****P < 0.0001; ns, not significant.

To evaluate the infectivity of the H10 isolates, Calu-3 cells were infected with the indicated virus at a multiplicity of infection (MOI) of 1.0 for a single-cycle infectivity assay. At 8 h post-infection (hpi), infected cells underwent immunofluorescence assays (IFAs) using an influenza nucleocapsid protein antibody. As shown in Fig. 2A and B, human isolate ZJ/2023 (H10N5) and wild bird isolate WB/2024 (H10N5) effectively infected Calu-3 cells. The average percentages of nucleoprotein (NP)-positive cells were 20.4% and 38.3%, respectively. Duck-derived isolate DK/2013 (H10N8) also effectively infected Calu-3 cells. The WB/2024 (H10N5) had a significantly higher infection rate than that of cells infected with the other two H10 viruses.

To determine whether the replication of the novel H10N5 virus in mammalian cells depended on L-1-tosylamide-2-phenylmethyl chloromethyl ketone (TPCK)-treated trypsin, Madin-Darby canine kidney (MDCK) cells were infected with the indicated virus at an MOI of 0.01 and incubated in a medium with or without TPCK-treated trypsin. Growth kinetics analysis showed that all three H10 viruses exhibited robust replication in the presence of trypsin, with the highest peak viral titer at approximately 10^7.75^ 50% tissue culture infective dose (TCID_50_)/mL (Fig. 2C). In contrast, in the absence of TPCK-treated trypsin, all three H10 viruses exhibited negligible replication, indicating that their replication depended on TPCK-treated trypsin, which is consistent with the hallmark feature universally shared by low-pathogenic avian influenza viruses.

To evaluate viral growth kinetics, Calu-3, A549, and DF-1 cells were infected at an MOI of 0.01 (Fig. 2D through F). In Calu-3 cells, the WB/2024 (H10N5), ZJ/2023 (H10N5), and DK/2013 (H10N8) viruses each reached their respective peak titers at 48 hpi, with titers of 10^5.63^, 10^7.0^, and 10^7.2^ TCID_50_/mL, respectively. The replication titer of WB/2024 (H10N5) was higher than that of ZJ/2023 (H10N5) at 12 hpi, which was consistent with IFA results. Immunofluorescence analysis of NP localization revealed that the nuclear import and/or export of NP protein occurred more rapidly in cells infected with WB/2024 (H10N5) compared to those infected with ZJ/2023 (H10N5) (Fig. S1). However, after 24 hpi, the replication rate of WB/2024 (H10N5) slowed, and ZJ/2023 (H10N5) and DK/2013 (H10N8) exhibited higher replication titers than WB/2024 (H10N5) after 48 hpi. DK/2013 (H10N8) reached higher replication titers than ZJ/2023 (H10N5) at 12–36 hpi, but their replication rates were similar at later stages. In A549 cells, the wild bird isolate WB/2024 (H10N5) reached its peak titer within 24 hpi at 10^5.54^ TCID_50_/mL, whereas ZJ/2023 (H10N5) and DK/2013 (H10N8) reached peak titers at 72 and 48 hpi, respectively. In DF-1 cells, ZJ/2023 (H10N5), WB/2024 (H10N5), and DK/2013 (H10N8) reached peak titers at 36 hpi, with titers of 10^6.04^, 10^4.25^, and 10^5.58^ TCID_50_/mL, respectively. After 24 hpi, ZJ/2023 (H10N5) and DK/2013 (H10N8) exhibited higher replication titers than WB/2024 (H10N5). These results suggest that, albeit with different infection kinetics, human and wild bird-derived H10N5 viruses can infect human respiratory epithelial cells and possess strong growth potential. In Calu-3 cells, all three H10 viruses reached peak titers at 48 hpi, consistent with previously reported human pandemic strains H1N1 and H3N2 (17). In A549 cells, the human H10N5 virus exhibited a replication kinetics profile similar to that of 2009 pandemic H1N1 viruses (18), with peak titers observed between 48 and 72 hpi.

### High titer of the novel H10N5 is lethal to BALB/c mice

**Fig 3:**
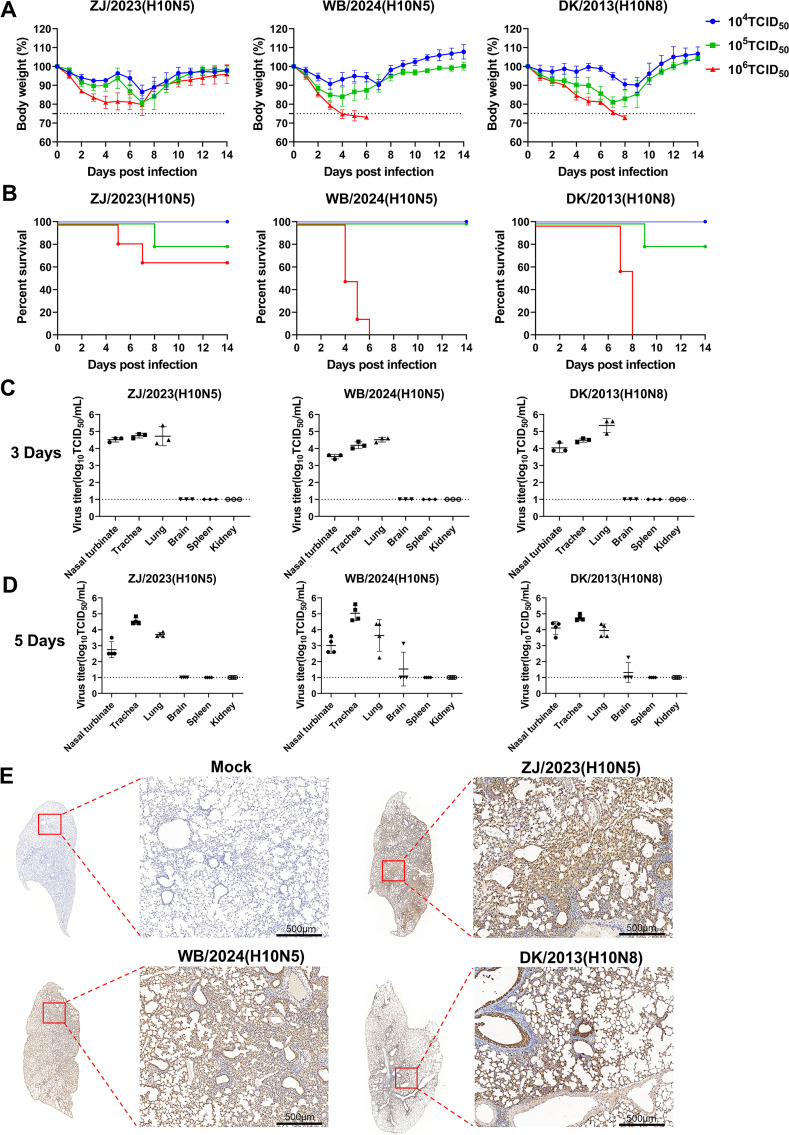
Pathogenicity and replication of novel H10N5 viruses in BALB/c mice. (**A and B**) Groups of six BALB/c mice were inoculated intranasally with 10^4^–10^6^ TCID_50_ of each virus indicated; weight change and survival were then monitored for 14 days. The dashed lines indicate 75% of the initial weight. (**C and D**) Mice were inoculated with the indicated virus at a dose of 10^6^ TCID_50_. At 3 and 5 days post-infection, groups of three and four mice were euthanized, respectively. Nasal turbinate, trachea, lung, brain, spleen, and kidney tissues were collected for virus titration by TCID_50_ assay on MDCK cells. The dashed lines indicate the lower limit of detection. (**E**) Representative immunohistochemical staining of mice lungs infected with the indicated viruses at 3 dpi. Lung tissue cells were stained by immunohistochemistry against influenza A virus nucleoprotein. All scale bars = 500  µm.

To evaluate the pathogenicity of novel H10N5 isolates in mammals, BALB/c mice were inoculated intranasally with 10^4^–10^6^ TCID_50_ of each virus and monitored for 14 days. Mice that lost more than 25% of their initial body weight were recorded as deaths and were sacrificed. As shown in Fig. 3A and B, after viral infection, mice exhibited significant clinical symptoms, including decreased appetite, reduced activity, fur ruffling, and a notable loss of body weight. At a dose of 10^4^ TCID_50_, all BALB/c mice infected with the three H10 viruses showed a weight loss exceeding 10% of their initial body weight. At a higher dose of 10^6^ TCID_50_, mortality occurred in mice infected with the human isolate ZJ/2023 (H10N5) and wild bird isolate WB/2024 (H10N5) at 5 and 4 days post-infection (dpi), respectively. This indicates that these two novel H10N5 viruses caused death after infecting mice with high titers. The mortality rate of WB/2024 (H10N5) reached 100% at an infective dose of 10^6^ TCID_50_.

To evaluate novel H10N5 replication in mice, mice were inoculated intranasally with 10^6^ TCID_50_ of each virus. Nasal turbinate, trachea, lung, brain, spleen, and kidney tissues were then collected for viral titration at 3 and 5 dpi, respectively. As shown in Fig. 3C and D, all three H10 viruses effectively replicated in mice nasal turbinate, trachea, and lung tissues without prior adaptation. Both WB/2024 (H10N5) and DK/2013 (H10N8) were detected in the brains of infected mice at 5 dpi, indicating the virus can cross the blood-brain barrier and cause tissue infections beyond that in the respiratory system. Pathological studies of lung tissues of infected mice revealed severe bronchiolitis and bronchopneumonia in all three H10-infected groups, characterized by alveolar septa thickening, edema, and interstitial inflammatory cell infiltration (Fig. S2A). The pathological change scores of ZJ/2023 (H10N5) and WB/2024 (H10N5) virus-infected lungs reached 4, whereas DK/2013 (H10N8) scored a maximum of 2 (Fig. S2B). Additionally, immunohistochemical (IHC) analysis was performed to detect viral antigens in the lung tissues of infected mice. This result revealed that the percentage of alveolar and bronchial epithelial cells positive for lung viral antigens in the three H10-infected mice exceeded 20%. The immunohistochemical scores were uniformly 4 (Fig. 3E; Fig. S2C), which is consistent with the high viral load observed in the lungs (Fig. 3C).

**Fig 4:**
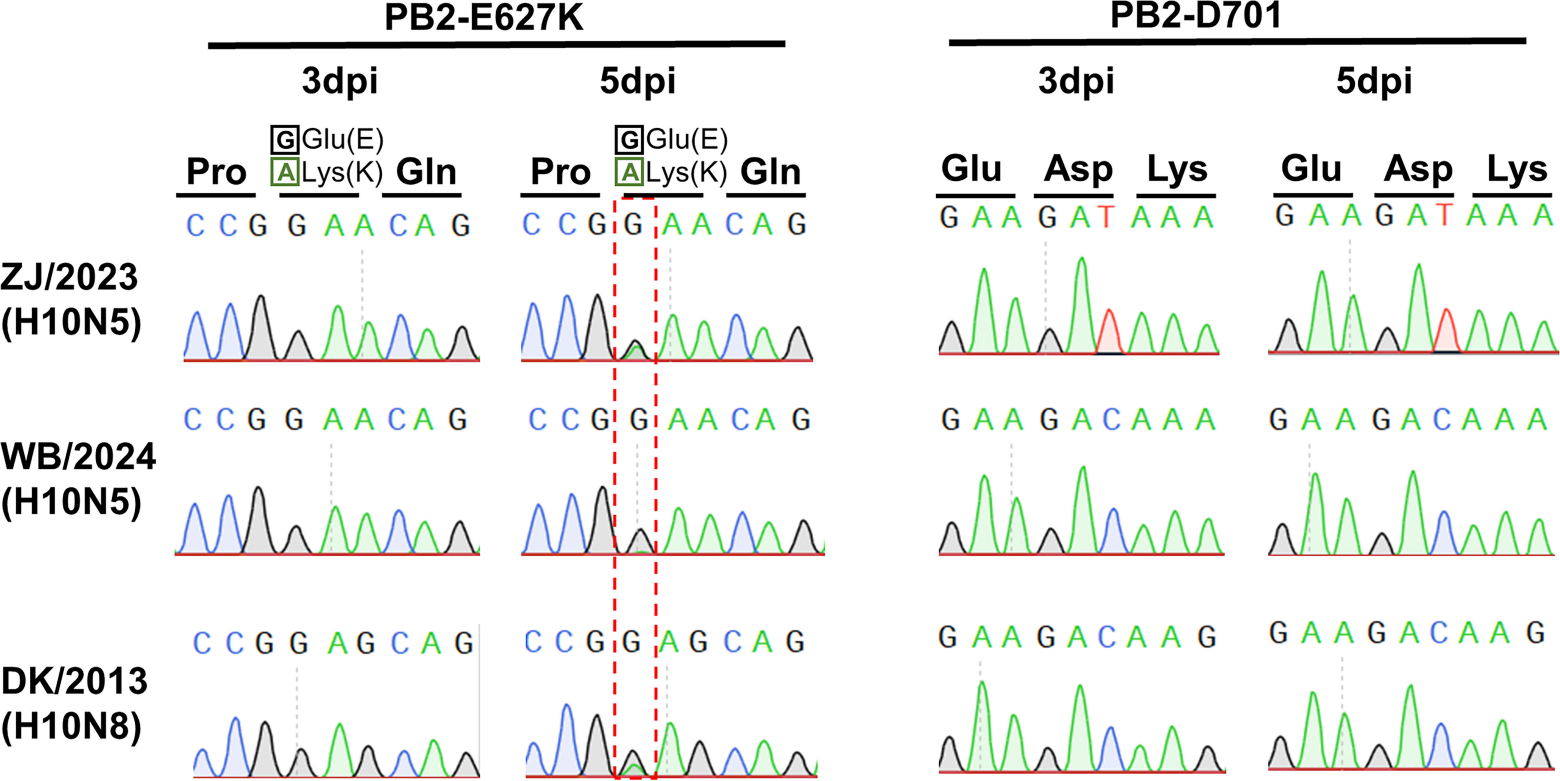
Changes at the PB2-E627K and PB2-D701N mutation sites in mice infected with the novel H10N5 virus. Sanger sequencing chromatograms were used to analyze the amino acid residues at the PB2 protein of viruses from mice lung tissues at 3 and 5 dpi with the three parental H10 viruses. Red dashed boxes indicate nucleotide substitutions at the corresponding positions within the PB2 protein. The results shown were obtained from sequencing pooled lung homogenates of three mice at 3 and 5 dpi, respectively.

All three H10 parental viruses possess glutamic acid at position 627 (Glu627) and aspartic acid at position 701 (Asp701) in the PB2 protein—two residues frequently associated with heightened virulence and improved adaptation in murine models when mutated to lysine (E627K) and asparagine (D701N), respectively (19, 20). To investigate the potential emergence of these adaptive mutations, we performed Sanger sequencing of lung tissues collected from infected mice at 3 and 5 dpi. The results revealed the appearance of the PB2 E627K mutation emerged as early as 5 dpi in the first passage of mice infected with the wild-type virus, whereas the PB2 D701N mutation was not detected (Fig. 4). Notably, the human lethal H10N8 virus isolated in 2013 harbored Lys at PB2 position 627, while other H10 avian influenza viruses that have infected humans retained the Glu at this site. These findings suggest that the H10N5 virus is capable of rapidly acquiring mammalian-adaptive mutations, further underscoring its potential threat to mammalian hosts.

### Analysis of pulmonary cytokines induced by high-dose H10N5 virus infection

**Fig 5:**
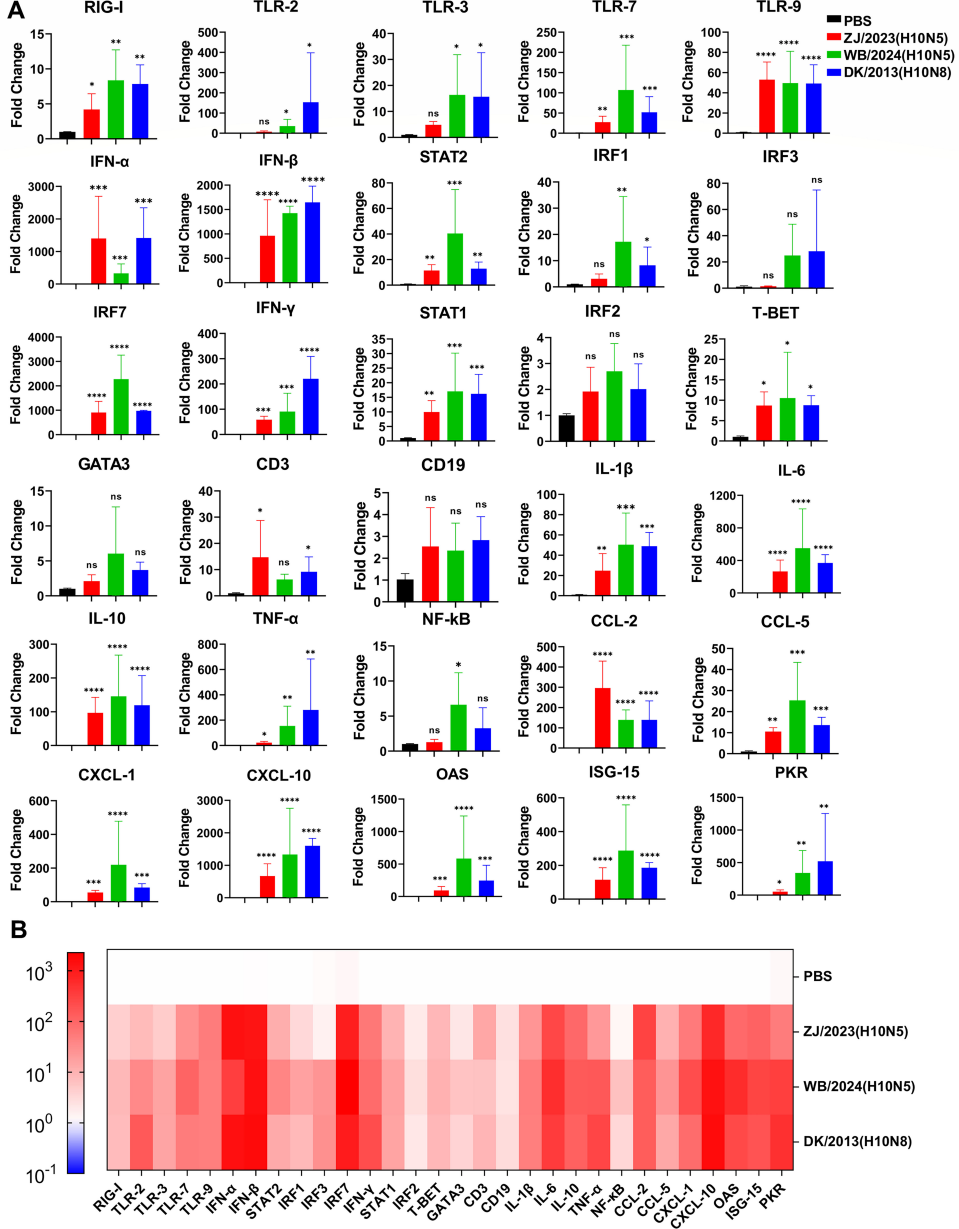
High-dose H10N5 virus challenge induces a cytokine storm in the lung tissues of BALB/c mice. (**A**) Relative mRNA expression levels of cytokines in the lung tissues of mice at 3 dpi. (**B**) Heat map of relative mRNA expression levels of cytokines. The test genes include pattern recognition receptors (RIG-1, TLR-2, TLR-3, TLR-7, and TLR-9), type I interferon response (IFN-α, IFN-β, STAT2, IRF1, IRF3, and IRF7), type II/general interferon response (IFN-γ, STAT1, and IRF2), TH1/2 response (T-BET and GATA3), T/B cell (CD3 and CD19), inflammatory cytokines (IL-1β, IL-6, IL-10, TNF-α, and NF-κB), chemokines (CCL-2, CCL-5, CXCL-1, and CXCL-10), and interferon-stimulated genes (OAS, ISG-15, and PKR). Primers were designed or acquired specific to *Mus musculus* genes (Table S3). The fold change of mRNA expression was calculated using the 2^−ΔΔCT^ method against the PBS group with GAPDH as the housekeeping gene. Each data point was assessed in triplicate. Statistical significance was calculated using log 10 values of fold change based on one-way ANOVA, compared with the corresponding value of the PBS group. *P < 0.05, **P < 0.01, ***P < 0.001, ****P < 0.0001. ns, not significant.

Cytokines play a crucial role in the viral clearance in the immune system. However, excessive cytokine production can cause severe immunopathology. Death caused by influenza is usually related to cytokine storms (21). To further investigate the mechanism of pulmonary immune damage induced by high-dose H10N5 infection, lung tissues from mice infected with 10^6^ TCID_50_ of the virus were collected at 3 dpi for host gene expression analysis. As shown in Fig. 5, the mice exhibited severe cytokine responses, characterized by significantly elevated levels of interferon (IFN), tumor necrosis factor (TNF), interleukin (IL), chemokines, and pro-inflammatory cytokines. These were detected in the lung tissues of the mice following severe viral infection. Compared to the phosphate-buffered saline (PBS) group, IFN-α and IFN-β in the lung tissue of mice infected with H10N5 were upregulated by hundreds to thousands of times. Pattern recognition receptors (PRRs), including RIG-I and TLR-2, were significantly upregulated (fold change >5 compared to the PBS group). TNF-α, IL-1, and IL-6, which are key pro-inflammatory cytokines released by the host during viral infection, were significantly upregulated (fold change >20 compared to the PBS group) (22). Additionally, the chemokines, including CCL2, CXCL1, and CXCL10, were upregulated several hundredfold. These cytokine and chemokine expression patterns resemble those observed in the lungs of mice infected with the mouse-adapted PR8 strain at 3 dpi (Fig. S3). Such a massive release of cytokines contributes to heightened inflammation and ultimately triggers a cytokine storm.

### Novel H10N5 cannot transmit in the guinea pig model

Guinea pigs are highly susceptible to influenza viruses and can transmit them to other guinea pigs through direct contact or aerosolized droplets. Consequently, they are commonly used as a model to evaluate the transmission of human influenza viruses (23, 24). In this study, we used a guinea pig model to investigate the horizontal transmission of the novel H10N5 virus, through direct contact and aerosol transmission.

Guinea pigs were infected with the indicated ZJ/2023 (H10N5), WB/2024 (H10N5), and DK/2013 (H10N8) at a dose of 10^6^ TCID_50_. The human H1N1pdm virus, XH25/2019 (H1N1pdm), confirmed to have transmission capability, served as a positive control. At 3 dpi, nasal wash, lung, brain, spleen, and kidney tissues were collected for viral titration. ZJ/2023 (H10N5) and WB/2024 (H10N5) replicated in the upper and lower respiratory tracts of the guinea pigs, whereas DK/2013 (H10N8) and XH25/2019 (H1N1pdm) were restricted to the upper respiratory tract. The DK/2013 (H10N8) titer in nasal washes was higher than those of ZJ/2023 (H10N5) and WB/2024 (H10N5) in nasal washes (Fig. S4A). Pathological analysis revealed that the lungs of guinea pigs infected with ZJ/2023 (H10N5) and WB/2024 (H10N5) exhibited extensive histopathological damage. This damage included lung pulmonary consolidation, bronchiolitis, and bronchopneumonia characterized by alveolar septum thickening and inflammatory cell infiltration. Conversely, few or no histopathological abnormalities were found in guinea pigs infected with DK/2013 (H10N8) (Fig. S4B). Viral NP antigen staining of lung tissues showed that >20% of cells in the lungs of guinea pigs infected with ZJ/2023 (H10N5) and WB/2024 (H10N5) were NP antigen positive. The immunohistochemical score was 4. In contrast, NP antigen-positive cells were rarely detected in the lungs of DK/2013 (H10N8)-infected guinea pigs (Fig. S4C and D).

**Fig 6:**
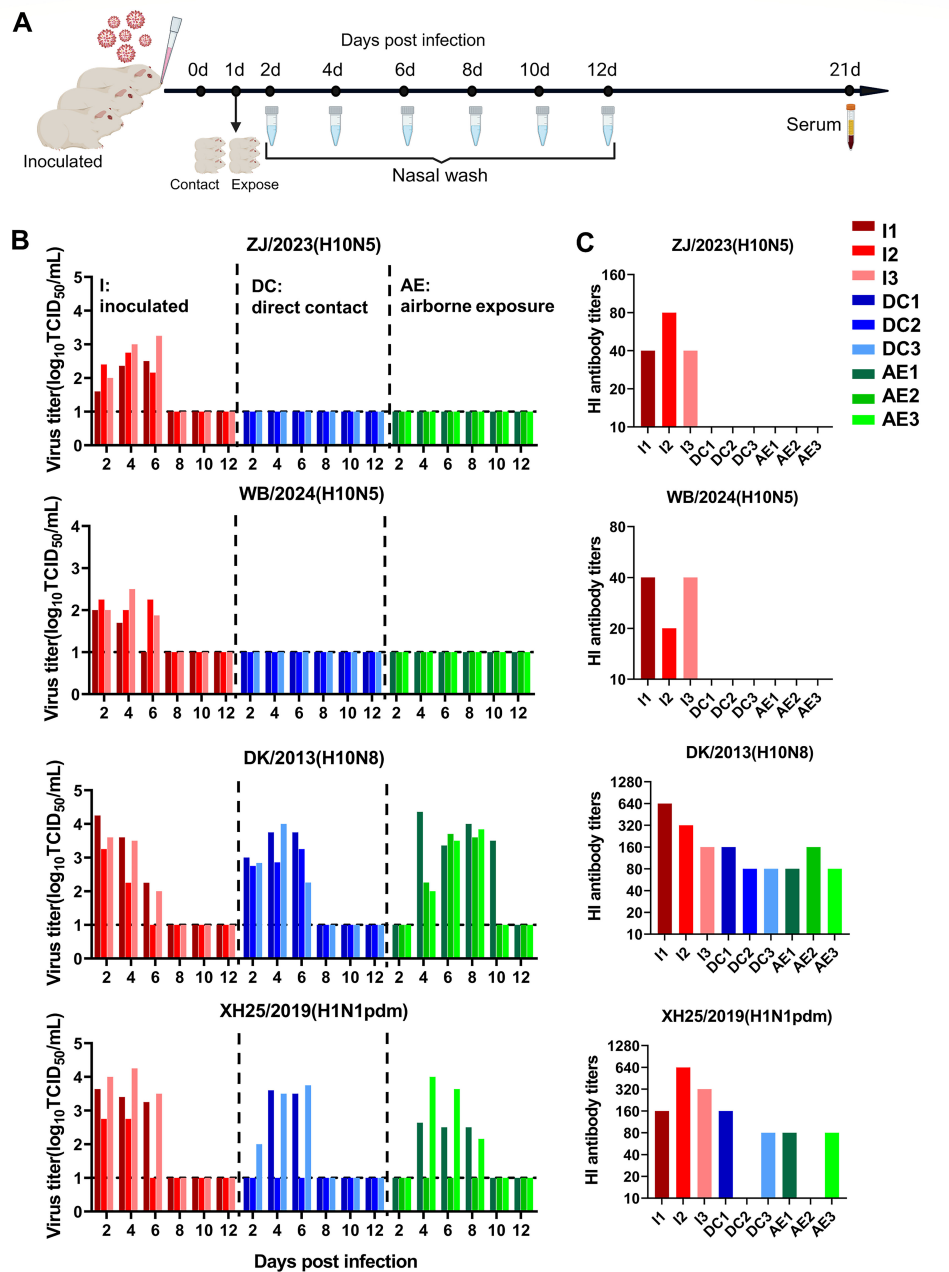
Horizontal transmission of novel H10N5 viruses in guinea pigs. (**A**) Diagram of the experimental procedure. Briefly, groups of three guinea pigs were inoculated intranasally with 10^6^ TCID_50_ of each virus indicated, including human H1N1pdm virus (XH25/2019) as control. Next day, three direct-contact guinea pigs were housed in the same cage as the inoculated guinea pig. At the same time, airborne-exposed guinea pigs were introduced into the other side of the cage (see also Fig. S2). Nasal washes for virus shedding detection were collected every 2 days after initial infection from all guinea pigs, continuing until 12 dpi, then titrated on MDCK cells. Seroconversion was analyzed by HI assay at 21 dpi. (**B**) The viral titers of virus shedding were assessed for each guinea pig during the transmission experiment. (**C**) HI antibody titers of virus-inoculated, direct-contacted, or exposed guinea pigs. AE, airborne exposed; DC, direct contact; I, inoculated. Each color bar represents the virus titer in an individual animal. The dashed lines indicate the lower limit of detection.

In the transmission study, groups of three guinea pigs were inoculated intranasally with 10^6^ TCID_50_ of each virus indicated. The next day, three direct contact guinea pigs were placed in the same cage as the inoculated guinea pig. Simultaneously, airborne exposure guinea pigs were placed on the other side of the cage (Fig. S5). Nasal wash viral titers were assessed for each guinea pig at 2 day intervals following the initial infection, and seroconversion was tested using the hemagglutination inhibition (HI) assay at 21 dpi (Fig. 6A). The virus was detected from nasal washes of ZJ/2023 (H10N5)-infected guinea pigs from 2 to 6 dpi, with titers ranging from 10^1.6^ to 10^3.25^ TCID_50_/mL. WB/2024 (H10N5)-infected guinea pigs exhibited a similar shedding pattern, though one guinea pig ceased shedding at 4 dpi. All inoculated guinea pigs seroconverted at 21 dpi. However, in guinea pigs infected with ZJ/2023 (H10N5) or WB/2024 (H10N5), no detectable viral shedding was observed in either the direct contact or airborne exposure groups. This suggests that these two viruses cannot currently be transmitted between guinea pigs. In contrast, in DK/2013 (H10N8)-infected guinea pigs, viral shedding was detected in all animals (including the inoculation, contact, and airborne exposure groups), with the highest titers of approximately 10^4.36^ TCID_50_/mL. Additionally, one guinea pig in the airborne exposure group continued shedding until 10 dpi, and all animals seroconverted. As a positive control, the human H1N1pdm virus could be transmitted via airborne exposure (Fig. 6B and C). H10 strains exhibited distinct tissue tropisms in guinea pigs. ZJ/2023 (H10N5) and WB/2024 (H10N5) exhibited a preference for lung infection, whereas DK/2013 (H10N8) showed a propensity to infect the upper respiratory tract. While ZJ/2023 (H10N5) and WB/2024 (H10N5) were capable of inducing severe pneumonia in guinea pigs, they could not transmit between them.

### Novel H10N5 exhibits dual receptor-binding properties

**Fig 7:**
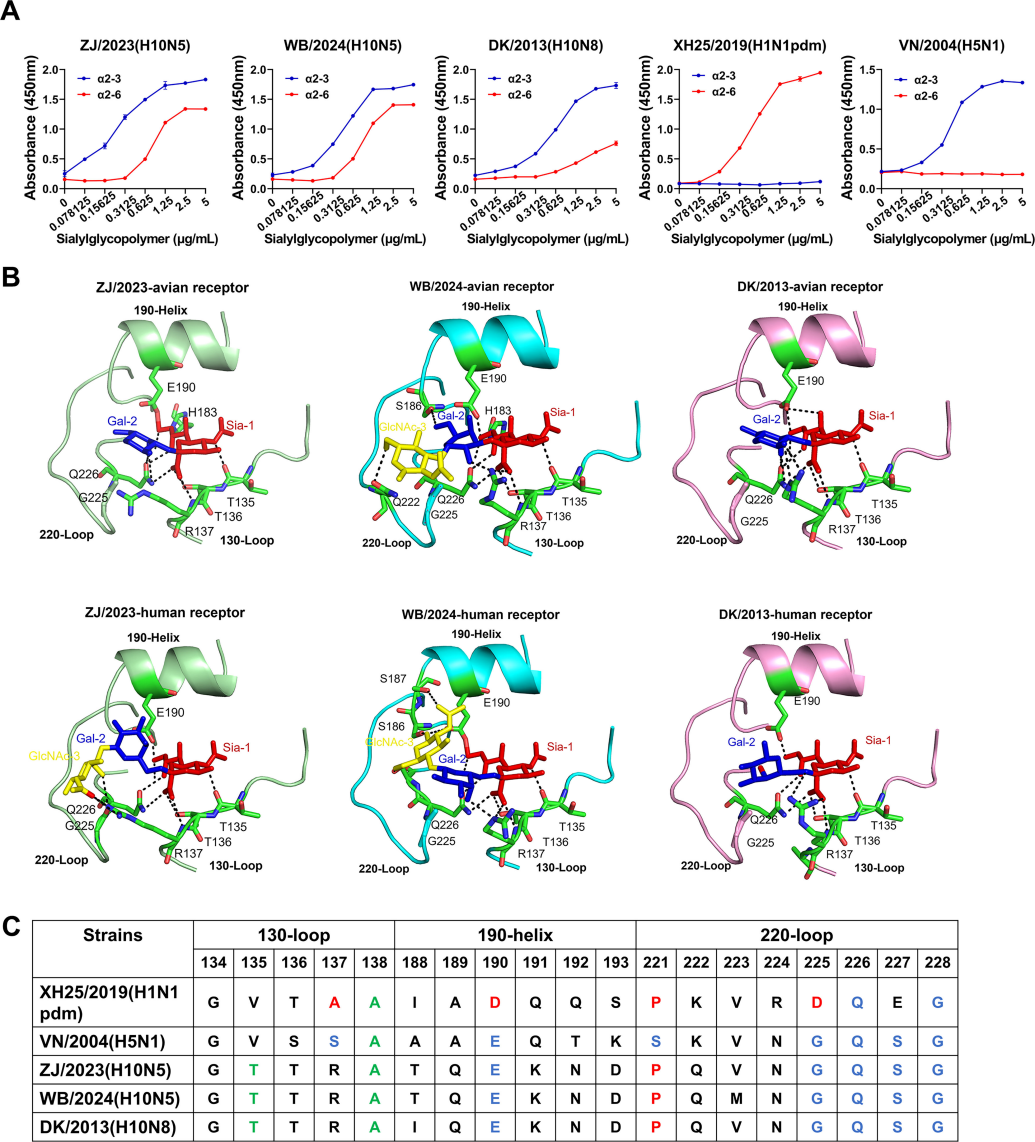
Receptor-binding properties and homology-modeling structural analysis of novel H10N5 virus. (**A**) Solid-phase binding assays for ZJ/2023 (H10N5), WB/2024 (H10N5), DK/2013 (H10N8), XH25/2019 (H1N1pdm), and VN/2004 (H5N1) viruses to both α2,3-linked (3′SLNLN) or α2,6-linked (6′SLNLN) sialic acid receptors. Binding to 3′SLNLN is marked in blue, and binding to 6′SLNLN is marked in red. ZJ/2023 (H10N5), WB/2024 (H10N5), and DK/2013 (H10N8) bind to both 3′SLNLN and 6′SLNLN. As a control, XH25/2019 (H1N1pdm) specifically binds 6′LNLN, and VN/2004 (H5N1) specifically binds 3′LNLN. The data shown consist of three independent replicates and are presented as mean ± standard deviation. (**B**) Structural analyses of the interactions of H10 AIV with either avian or human receptor analogs. PyMOL software was used to visualize hydrogen bonds between amino acid residues and the glycan receptors. The three secondary structural elements of the binding site (130 loop, 190 helix, and 220 loop) are labeled in cartoon representation, together with specific residues in stick representation. The red part of the glycan receptor represents Sia-1; the blue part represents Gal-2; and the yellow part represents GlcNAc-3. (**C**) Sequence alignment of the key residues in the receptor-binding site region of the hemagglutinins. The green markers represent key amino acids associated with mammalian adaptation; red represents amino acids associated with human-like receptor binding; and blue represents amino acids associated with avian-like receptors.

The binding ability of the HA protein to human-type receptors is a key factor in viral adaptation in humans. Therefore, we characterized the receptor-binding ability of the human isolate ZJ/2023 (H10N5) and wild bird isolate WB/2024 (H10N5) and compared them with that of the duck isolate DK/2013 (H10N8) AIV. As controls, we included A/Wuhan/XH25/2019 (H1N1pdm) and A/Vietnam/1194/2004 (H5N1), known for their high affinity for α2,6-linked sialic acid (human-type receptor) and α2,3-linked sialic acid (avian-type receptor), respectively. A solid-phase binding assay (Fig. 7A) showed that most H10 AIV isolates are preferentially bound to avian-type receptors. The human-derived H10N5 and wild bird-derived H10N5 viruses exhibited the ability to bind to dual receptors, SAα-2,3-Gal and SAα-2,6-Gal, with slightly lower affinity for SAα-2,6-Gal than for SAα-2,3Gal. Additionally, ZJ/2023 (H10N5) and WB/2024 (H10N5) showed a higher binding affinity for SAα-2,6-Gal than for DK/2013 (H10N8).

Further analysis of key residues at the receptor-binding site revealed that ZJ/2023 (H10N5) and WB/2024 (H10N5) retained the characteristics of avian influenza virus receptor-binding sites (Glu190, Gln226, and Gly228). However, the receptor-binding properties of the virus were largely determined by the overall conformation of the 220 loop of HA. The Gly225 residue may alter the conformational flexibility of the 220 loop, enabling dual receptor-binding properties (25). Previous studies show that Glu190 and Gly225 reshape the 220 loop, allowing Gln226 to move closer to Gal-2 by forming a water-mediated hydrogen bond network between Glu190 and Gln226 (26). Molecular docking revealed that Sia-1 in human glycan receptors formed extensive potential hydrogen bonds with Thr135 and the adjacent Thr136 and Arg137 on the 130 loop, facilitating the binding of the viruses to human-type receptors (Fig. 7B). Additionally, the HA of these viruses acquired a human-specific mutation (Pro221) in the 220 loop (Fig. 7C). The Pro221 residue may enhance the affinity of avian influenza viruses for SAα-2,6-Gal (27). These findings suggest that H10N5 viruses have acquired key mutations that enhance their ability to bind to human receptors, posing a significant risk of crossing the species barrier and infecting humans.

## DISCUSSION

H10 subtype AIVs infect poultry and have been circulating in live poultry markets in China for years (28, 29). Wild birds are the natural hosts and reservoirs of AIV, and their migration is believed to play a key role in the emergence of novel AIV in humans (30, 31). Reassortment of AIVs in poultry and wild birds facilitates cross-species transmission and infection of humans (32, 33). Findings suggest that the novel avian influenza A H10N8 virus, which causes human infection and death, was established through multiple reassortment events within poultry and wild birds (34). Similarly, the human H10N5 virus is a novel reassortant that originated from wild bird influenza viruses and sporadically infected humans (35). In this study, we demonstrated that human H10N5 and wild bird H10N5 exhibited significant replication in human lung epithelial cells and lethal virulence in mammalian hosts and displayed a high affinity for human receptors. These characteristics highlight their potential to infect humans. However, neither H10N5 strain was transmissible between guinea pigs, indicating they have not yet acquired the ability to spread effectively.

Receptor-binding affinity and host adaptation are critical for the replication and transmission of influenza viruses. HA is a viral surface glycoprotein responsible for viral entry into host cells. It achieves this by binding to sialic acid receptors on the cell surface, followed by pH-triggered membrane fusion within the endosomal compartment. The shift in receptor-binding specificity from avian α2-3- to human α2-6-linked receptors is a crucial prerequisite for influenza A virus to overcome species barriers, adapt to new hosts, and spread in humans (36). In this study, Glu190, Gln226, and Gly228 (H3 numbering) in the receptor-binding site of human H10N5 HA and wild bird H10N5 HA suggest a preference for avian-like receptors. However, they also exhibit high-affinity binding to human-like receptors, demonstrating a dual receptor-binding profile. Previous research has suggested that Ser138Ala of the HA gene may be associated with increased binding affinity to human α2,6-linked sialic acid receptors (37). Interestingly, our receptor-binding results for the ZJ/2023 (H10N5) virus differ from those reported in earlier studies (10), despite the use of the same assay methodology. This discrepancy may stem from differences in the origin and specificity of the detection antibodies rather than from any intrinsic alterations in the receptor-binding properties of the virus itself. Therefore, further comparative studies using matched reagents and standardized controls may help to clarify these findings. Additionally, molecular docking analysis of the HA protein and the polysaccharide receptor reveals that Sia-1 in the human glycan receptor forms extensive potential hydrogen bonds with certain amino acids in the 130 loop, promoting virus binding to human receptors.

Pathogenicity experiments indicated that novel H10N5 was lethal to mice and efficiently replicated in the nasal turbinate, trachea, and lungs. This is consistent with previous research findings that the human H10 viruses exhibit robust pathogenicity in BALB/c mice (38, 39) and that migratory wild bird H10 viruses can replicate in mice, causing significant clinical symptoms without prior adaptation (16). Despite lacking multiple basic amino acid motifs at the HA cleavage site [PEVVQGR↓G for ZJ/2023 (H10N5) and WB/2024 (H10N5), PELIQGR↓G for DK/2013 (H10N8)], the two H10N5 demonstrated significant replication capabilities both *in vivo* and *in vitro*. The H10N5 viral genome displayed some genetic features related to mammalian adaptation and virulence. HA contains Ala135Thr and Ser138Ala substitutions (H3 numbering), which facilitate adaptation to mammals (40). Additionally, substitutions Asn30Asp, Thr215Ala in M1, and Pro42Ser in NS1 are associated with increased virulence in mice (9, 13).

In a study of host responses to high-dose H10N5 infection in mice, the levels of cytokines including PRR, IFN, TNF, IL, and chemokines increased in lung tissue significantly. PRR recognizes pathogen-associated molecular patterns, triggering immune signaling pathways and initiating inflammatory responses. IL-1 and IL-6 are key pro-inflammatory cytokines released by the host during viral infection (22). Unlike pro-inflammatory cytokines, chemokines usually exhibit specific chemotactic activities, directing the migration of monocytes and T lymphocytes to sites of inflammation (41). In this study, chemokines including CCL2, CXCL1, and CXCL10 were significantly upregulated. Notably, CCL2 and CXCL10 levels are abnormally elevated in certain fatal cases of influenza infection (42). Previous studies show that the chemokine receptor CCR2 (the primary receptor for CCL-2) is crucial in severe influenza infection (43). CCR2−/− mice showed a lower inflammatory response but significantly increased viral load. This evidence partially suggests that the pathology of severe influenza is mediated by cytokine response rather than viral load. In summary, high-dose H10N5 infection in mice induced a severe cytokine storm and excessive accumulation of inflammatory cells and exudate in the lungs. This led to severe pneumonia and subsequent hypoxia, a key factor leading to mouse death.

In guinea pig transmission experiments, both human-derived H10N5 and wild bird-derived H10N5 strains failed to transmit via direct contact. In contrast, the duck-derived H10N8 strain exhibited significant airborne transmission capability. The H10N8 strain used in this study carries internal genes of the H9N2 virus. Evidence suggests that H9N2 has facilitated the emergence and evolution of novel AIV capable of infecting humans, such as H3N8, H7N9, H10N8, and H5N6 (3, 44, 45). AIV with H9N2-derived internal genes may possess enhanced ability to infect humans. Consequently, poultry carrying H9N2 may serve as a gene reservoir for the generation of novel reassorted AIV (46). Thus, the reassortment of the novel H10N5 with avian H9N2 deserves high attention. We further investigated the amino acid sites that may influence airborne transmission. PB2 627K and 701N are known to facilitate influenza virus replication in the mammalian upper respiratory tract and are critical for efficient viral transmission (47–49). However, all three H10 viruses in this study possessed PB2 627E and 701D, suggesting that other amino acids may contribute to airborne transmission between guinea pigs. Comparative analysis revealed that DK/2013 (H10N8) harbors mutations including I292V, R389K, A588V, T598V, L648V, and T676M in PB2; I368V mutation in PB1; and A37S, I61T, V63I, A343S, K356R, and S409N mutations in PA (Table S2), which represent mammalian adaptive markers (50–52). The above mutations may acquire airborne transmission ability by promoting the adaptation in mammals and replication efficiency in the upper respiratory tract. Therefore, we should be vigilant about the sustained antigen drift of H10N5, which could lead to its circulation in human populations.

Taken together, this study provides a comprehensive analysis of the biological characteristics, mouse pathogenicity, transmissibility, and receptor-binding properties of the novel H10N5. These findings offer new insights into the prevalence of this potential zoonotic H10N5 and provide timely warnings and reports for the development of public health prevention strategies.

## MATERIALS AND METHODS

### Cells, viruses, and animals

Calu-3 cells were cultured in minimum essential medium supplemented with 10% fetal bovine serum (FBS). Human lung carcinoma cells (A549), MDCK, and chicken embryo fibroblasts (DF-1) cells were cultured in Dulbecco’s Modified Eagle’s Medium supplemented with 10% FBS at 37°C and 5% CO_2_ in an incubator. Following viral infection, MDCK and Calu-3 cells were maintained in culture medium supplemented with 0.3% bovine serum albumin (BSA, Sigma-Aldrich) and 2 µg/mL TPCK-treated trypsin (T1426, Sigma-Aldrich). A549 and DF-1 cells were maintained in medium containing 0.3% BSA and 1 µg/mL TPCK-treated trypsin.

Based on published sequences of A/Zhejiang/CNIC-ZJU01/2023_H10N5 [ZJ/2023 (H10N5) and EPI_ISL_18846022], genes were synthesized by Sangon Biotech Company, and the virus was rescued by using reverse genetics (53). A/wild birds/Hubei/YYH40/2024_H10N5 [WB/2024 (H10N5) and EPI_ISL_19504791] was isolated from wild bird fecal samples in our laboratory and verified using next-generation sequencing. A/Duck/Jiangxi/NCQSH323/2013_H10N8 [DK (H10N8) and EPI_ISL_220159], A/Wuhan/XH25/2019_H1N1pdm [XH25/2019 (H1N1pdm) and EPI_ISL_19769179], and A/Vietnam/1194/2004_H5N1 [VN/2004 (H5N1) and EPI_ISL_11976295] were previously kept in our laboratory. All viruses were stored at −80°C after propagation for future use.

Specific pathogen-free (SPF) BALB/c mice and Hartley strain female guinea pigs were purchased from Beijing Vital River Laboratory Animal Technology Co., Ltd (Beijing, China). The animals were housed in SPF or animal biosafety level 2 facilities of the Animal Experiment Center at the Wuhan Institute of Virology, CAS, with adequate food and water.

### Phylogenetic analysis

H10 subtype HA gene sequences were obtained from GISAID (https://gisaid.org/). Subsequently, duplicate and low-quality sequences were removed, leaving 1,889 sequences. Multiple sequence alignments were performed using MAFFT software. Maximum likelihood was reconstructed separately with IQ-TREE2 using ultrafast bootstrap resampling analysis (1,000 replicates) and best-fit nucleotide substitution models. Phylogenetic trees were visualized and annotated using FigTree (version 1.4.4) and Adobe Illustrator 2022.

### Growth kinetics of viruses and TPCK-treated trypsin dependence in cell culture

Calu-3, A549, and DF-1 cells were cultured in 24-well cell culture plates. Infection was performed when the cells reached 90% confluence. The cells were gently washed thrice with warm PBS to remove excess culture medium before infection. After washing, each virus was inoculated at an MOI of 0.01, with three replicate wells per virus at each time point. After incubation for 2 h, the inoculum was removed, and the cells were washed thrice with PBS. The medium was then replaced with the cell maintenance medium. The resulting supernatants were subsequently collected at 12, 24, 36, 48, 60, 72, 84, and 96 hpi. Viral titers were determined using the TCID_50_ of the cells in MDCK cells. For the TPCK-treated trypsin-dependent assay, MDCK cells were infected with the indicated virus at an MOI of 0.01. The viral inoculums were removed after 1.5 h of adsorption and replaced with maintenance medium with or without TPCK-treated trypsin (2 µg/mL) at 37°C. The resulting supernatants were harvested at 12, 24, 48, and 72 hpi, and viral titers were determined using MDCK cells as described above.

### Immunofluorescence microscopy

Calu-3 cells were infected with viruses at an MOI of 1. After incubation for 2 h, the inoculum was removed and replaced with cell maintenance medium. At 8 hpi, the cells were fixed in 4% paraformaldehyde for 30 min. Then, the cells were permeabilized with 0.2% Triton X-100 (Beyotime, Beijing, China) at room temperature for 20 min and blocked with 2% BSA in phosphate-buffered saline with Tween 20 (PBST) for 2 h at 37°C. The cells were stained with influenza A virus nucleoprotein antibodies (GeneTex, GTX125989) overnight at 4°C. The cells were then washed five times with PBST and incubated with Alexa Fluor 568 goat antirabbit IgG secondary antibody (1:500, A11036; Invitrogen). To localize the cells, they were stained with Hoechst 33258 staining solution (C1018, Beyotime) in the dark for 5 min, washed four times with PBST, and mounted with Antifade Mounting Medium (P0126, Beyotime). The stained cells were imaged using a Leica STELLARIS 8 laser scanning confocal microscope (Leica, Germany). NP-positive cells were subsequently quantified using ImageJ software (Media Cybernetics, USA).

### Mouse challenge studies

To evaluate the pathogenicity of the novel H10N5 viruses to mammals, 6- to 8-week-old female BALB/c mice were used. Groups of six mice were anesthetized with avertin (240 mg/kg) and intranasally challenged with 50 µL virus at 10-fold serial dilutions from 10^4^ to 10^6^ TCID_50_. Clinical signs of infection and weight loss were monitored daily for 14 days post-challenge. Mice that lost 25% or more of their initial body weight were humanely euthanized.

To evaluate the viral replication in various tissues of mice, groups of mice were infected with 10^6^ TCID_50_ of the virus and euthanized at 3 and 5 dpi, respectively. Nasal turbinate, trachea, lung, brain, spleen, and kidney samples were collected for viral titration. At the same time, lung tissues from mice infected at 3 dpi were collected, fixed in 4% neutral-buffered formalin, embedded in paraffin, and cut into sections. The resulting tissue sections were then used for hematoxylin-eosin (HE) staining and immunohistochemistry staining analysis.

### Relative gene expression levels in lung tissue

RNA from the lung tissue supernatant of virus-infected mice was extracted using the tissue total RNA isolation kit (RC112, Vazyme) according to the manufacturer’s instructions. In all host genes, qRT-PCR was performed in triplicate using Taq Pro Universal SYBR qPCR Master Mix (Q712, Vazyme) on cDNA synthesized with HiScript III 1st Strand cDNA Synthesis Kit (R312, Vazyme). *Mus musculus* host gene-specific primers are shown in Table S3. The fold change of mRNA expression was calculated using the 2^−ΔΔCT^ method against the PBS group with GAPDH as the housekeeping gene.

### Replication and transmission in guinea pigs

Female Hartley guinea pigs weighing 250–350 g that were serologically negative for influenza viruses were used in this study. Guinea pigs were anesthetized by intramuscular injection of ketamine (20 mg/kg) and xylazine (1 mg/kg). To assess viral replication, three guinea pigs per group were intranasally infected with 10^6^ TCID_50_ of the virus, with a volume of 300 µL (150 µL per nostril). At 3 dpi, the guinea pigs were euthanized, and nasal washes, lung, brain, spleen, and kidney were collected for virus titration in MDCK cells. HE and IHC staining analyses were subsequently performed.

To evaluate direct contact transmission and respiratory droplet transmission of the virus, each group of animals was assigned a “1:1:1” with three pairs, conducted in three separate cages as shown in Fig. S5. One guinea pig was intranasally inoculated with 10^6^ TCID_50_ of the virus and placed in a specially designed cage. After 24 h, one naive guinea pig was placed together with the infected animal as the direct contact group. Another naive guinea pig was placed on the opposite side of the cage, separated by a double-layer mesh (5 cm apart). Airflow moved from the infected guinea pig toward the aerosol exposure group. Nasal washes were collected at 2 day intervals, beginning on 2 dpi (1 day post-exposure) and titrated in MDCK cells to assess viral shedding in the guinea pigs. Serum was collected from all animals at 21 dpi and treated with receptor-destroying enzyme for HI antibody testing to evaluate seroconversion.

### Receptor-binding assay

Receptor-binding specificity of the viruses was determined using a solid-phase direct binding assay as described previously (54, 55). Briefly, serial dilutions of biotinylated glycans 3′SLN-C3-PAA-biotin (Neu5Aca2-3Galb1-4GlcNAcb-sp3, 0036 BP, GlycoNZ) or 6′SLN-C3-PAA-biotin (Neu5Aca2-6Galb1-4GlcNAcb-sp3, 0997 BP, GlycoNZ) were prepared in PBS (5.0, 2.5, 1.25, 0.63, 0.31, 0.16, and 0.08 µg/mL). Diluted biotinylated glycans (50 µL) were added to the wells of the 96-well streptomycin-coated microplate (22351, BEAVERBIO) and incubated overnight at 4°C. After washing twice with PBS, the plate was blocked with 0.1 mL PBS containing 2% BSA at 25°C for 1 h, followed by three washes with PBS. The viral dilutions, containing 64 hemagglutinin units, were incubated with neuraminidase inhibitors at a final concentration of 100 nM (oseltamivir and zanamivir) at 37°C for half an hour, and then the viral solution was incubated with different concentrations of glycans overnight at 4°C. After washing with PBST, the plates were incubated with mouse antisera against H10N8 influenza virus and horseradish peroxidase-conjugated goat antimouse IgG (SA00001-1, Proteintech). A rabbit monoclonal antibody against H1N1 HA (ab281949, Abcam) and a rabbit polyclonal antibody against H5N8 HA (11717-RP02, Sino Biological) were used for the detection of H1N1 and H5N1 viruses, respectively. Then, the plates were washed five times, and 0.1 mL of 3,3,5,5′-tetramethylbenzidine was added to the plates as chromogenic substrates, followed by the addition of 0.1 mL of H_2_SO_4_ as the stop solution and substrate. The absorbance was measured at 450 nm.

### Homology modeling and molecular docking study

Homology modeling of the H10 HA protein was performed using AlphaFold2 as previously mentioned (56). Energy minimization molecular docking studies using the AutoDock Vina program were conducted to evaluate the potential interaction sites between glycan receptors and the HA protein. Water molecules were removed, and polar hydrogen was added to the target protein before the docking study. Following the docking procedure, 10 conformations for each target-ligand complex were generated, and the best conformation was chosen according to lowest negative energy. PyMOL software was used to visualize interactive residues between hemagglutinin and glycan receptor.

## SUPPLEMENTAL MATERIAL

Supplemental material mbio.00731-25-s0001.docx Fig. S1 to S5; Tables S1 to S3.

ASM does not own the copyrights to Supplemental Material that may be linked to, or accessed through, an article. The authors have granted ASM a non-exclusive, world-wide license to publish the Supplemental Material files. Please contact the corresponding author directly for reuse.
