# Supplementary material for: Pathogenicity, transmissibility, and receptor binding of a human-isolated influenza A (H10N5) virus
Source: mBio. 2025 Jul 8;16(8):e00731-25. doi: 10.1128/mbio.00731-25 (PMC12345265; doi:10.1128/mbio.00731-25)
Supplement: Supplemental material — Fig. S1 to S5; Tables S1 to S3. [file mbio.00731-25-s0001.docx]

**Supplementary Materials**

**Pathogenicity, transmissibility and receptor-binding of a human isolated influenza A (H10N5) virus**

Mengchan Hao,^1,2^ JiaYing Wu,^1,2^ LiNa Ji,^3^ Yubo Zhao,^1,2^ Shunyuan Zhang,^1,2^ Yiwei Guan,^1,2^ LiangYu Li,^4^ Wenxue Yang,^1^ Yuan Zhang,^*,1,2^ Jianjun Chen^*,1,2^

*^1^ State Key Laboratory of Virology and Biosafety, Wuhan Institute of Virology, Chinese Academy of Sciences, Wuhan 430071,* *People’s Republic of China;*

*^2^ University of Chinese Academy of Sciences, Beijing 100049, People’s Republic of China;*

*^3^ School of Life Sciences, Inner Mongolia University, Hohhot 010070, People’s Republic of China;*

*^4^ Department of Pulmonary and Critical Care Medicine, Renmin Hospital of Wuhan University, Wuhan 430060, People’s Republic of China.*

Running head: Potential risks assessment of novel H10N5 influenza virus

*corresponding author

Jianjun Chen

Email: chenjj@wh.iov.cn

Yuan Zhang

Email: zhangyuan@wh.iov.cn


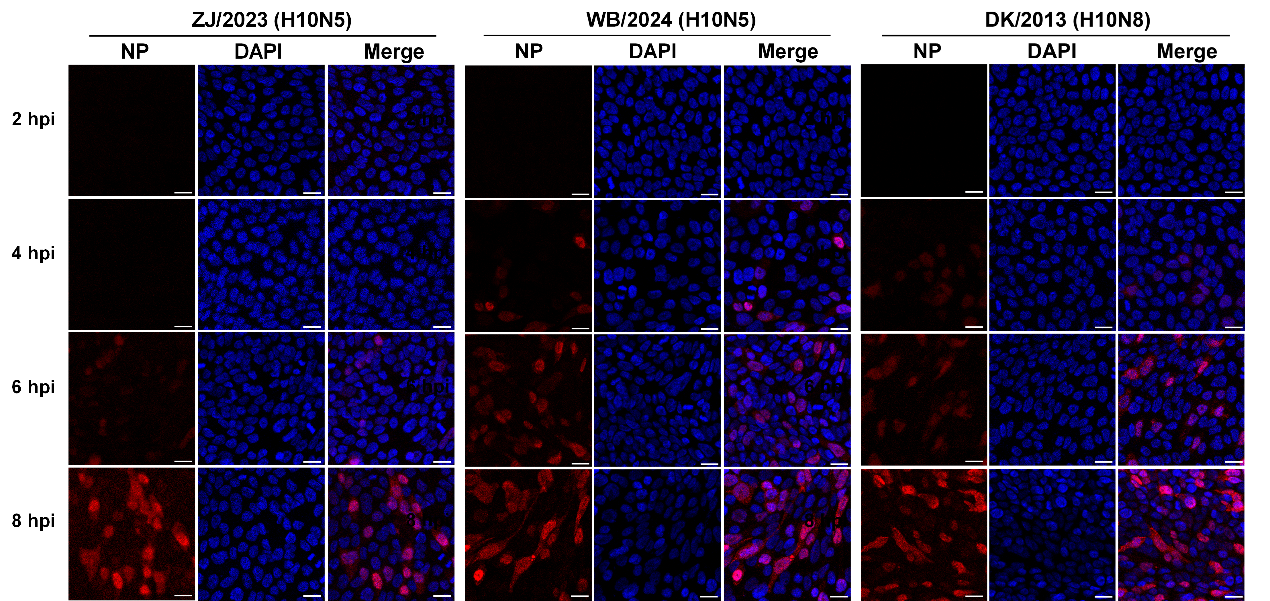


**Fig. S1.** Intracellular distribution of NP protein in Calu-3 cells infected with H10 viruses. Calu-3 cells were infected with the indicated H10 viruses at an MOI of 5.0. At 2, 4, 6, and 8 hours post-infection (hpi), cells were fixed with 4% paraformaldehyde (PFA) and stained with an anti-influenza nucleoprotein (NP) monoclonal antibody to detect intracellular NP localization. Cell nuclei were stained with Hoechst 33258. Merged images show the overlay of NP and nuclear signals. Scale bar: 20 µm.


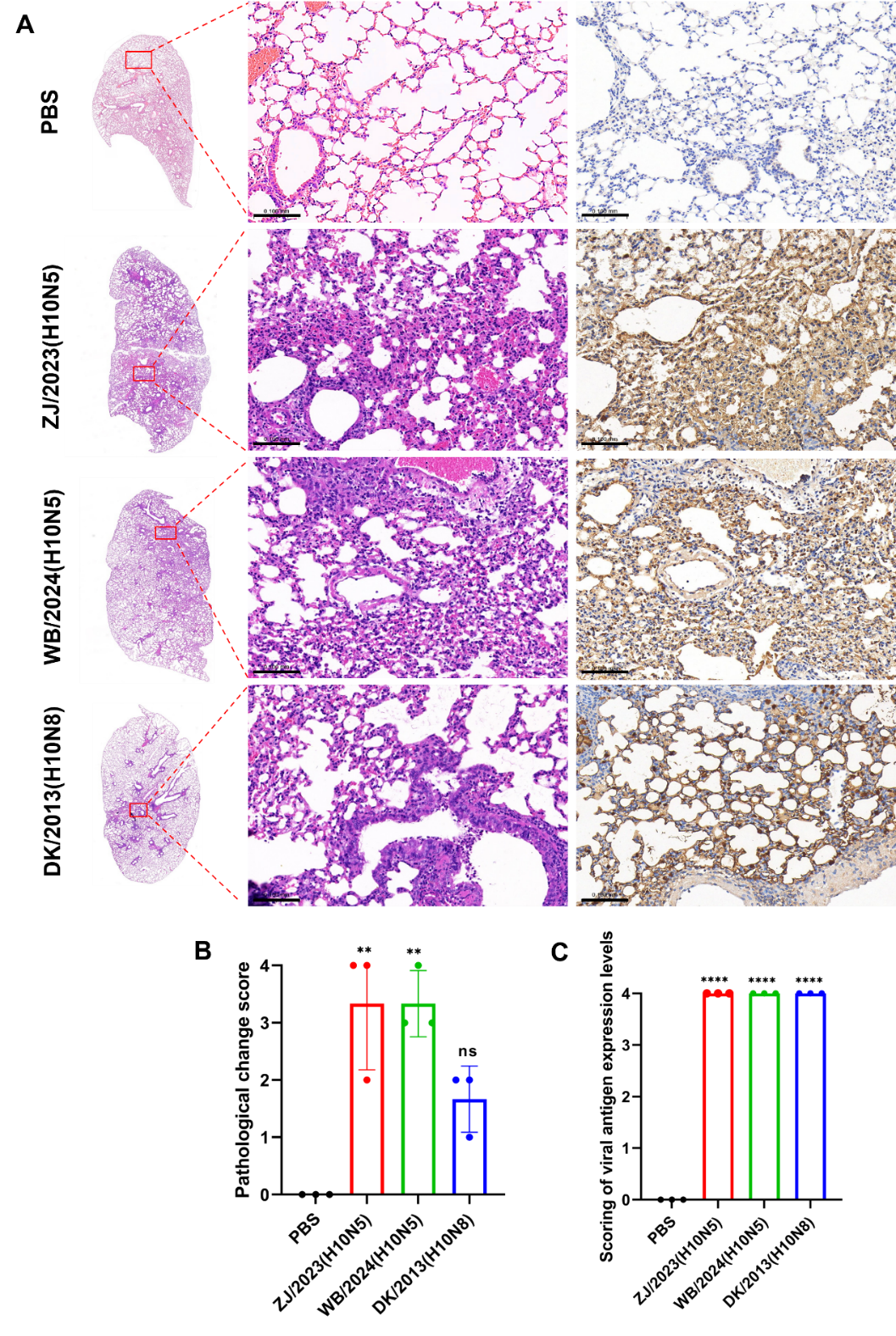


**Fig. S2.** Pathogenicity and replication of novel H10N5 viruses in lungs of BALB/c mice. Representative Hematoxylin-Eosin (HE) staining and immunohistochemistry (IHC) staining of the lungs of mice infected with the specified virus at 3 dpi (A). The pathological change score (B) and immunohistochemical scores (C) in the lungs of mice inoculated with indicated viruses at doses 10^6^ TCID_50_ at 3 dpi were performed. The pathological changes in lung tissues were scored based on the area of tissue damage: 0, no visible lesions; 1, affected area by the lesions (< 10%); 2, affected area by the lesions (< 30%, ≥ 10%); 3, affected area by the lesions (< 50%, ≥ 30%); 4, affected area by the lesions (≥ 50%). The immunohistochemical scoring standard was based on the percentage of virus antigen-positive cells: 0, no positive cells; 1, positive cells (< 5%); 2, positive cells (< 10%, ≥ 5%); 3, positive cells (< 20%, ≥10%); 4, positive cells (≥ 20%). All scale bars = 100 µm. Statistical significance was based on one-way ANOVA, compared with the corresponding value of PBS (* *p* < 0.05, ** *p* < 0.01, *** *p* < 0.001, **** *p* < 0.0001 and ns, not significant).


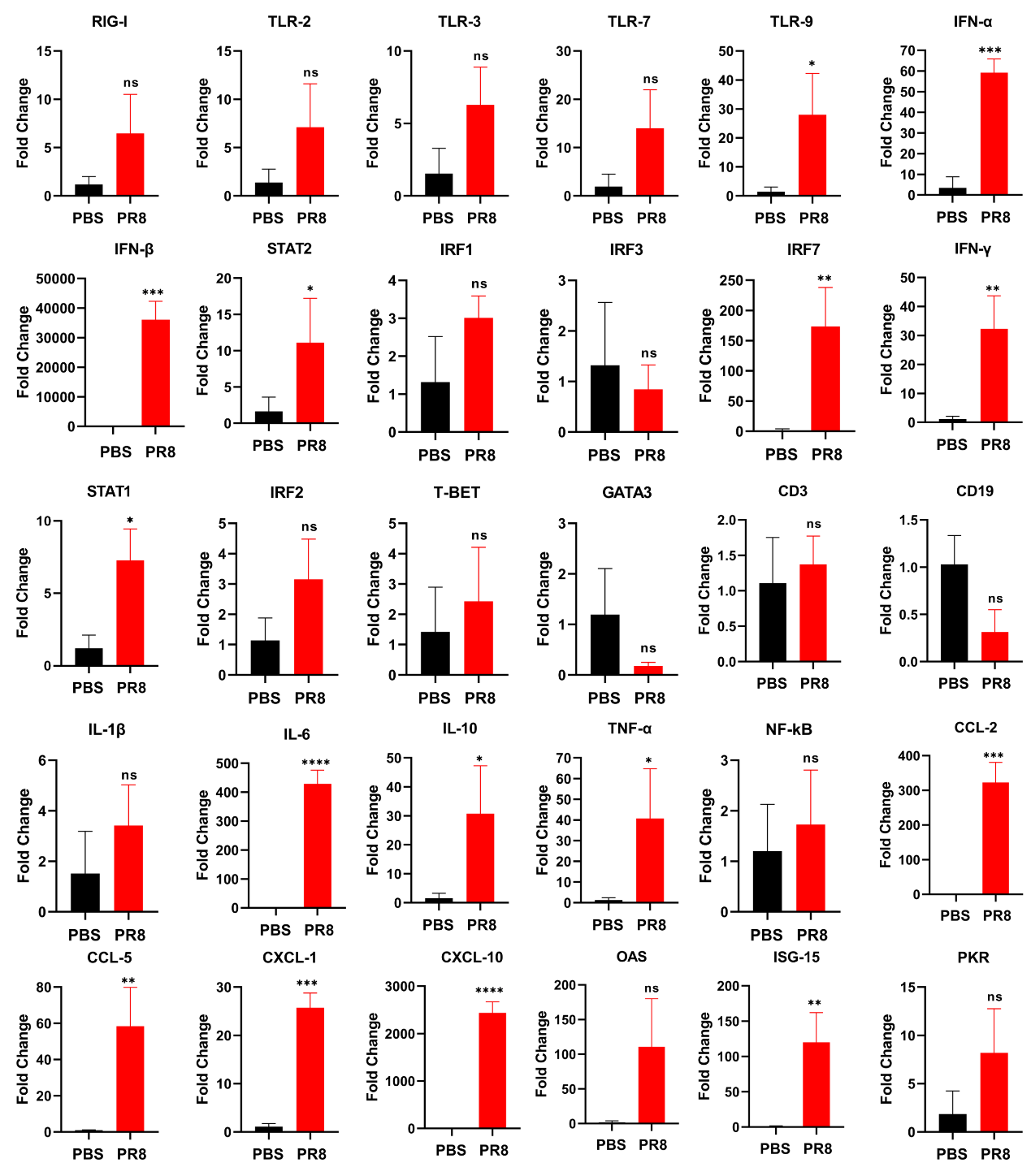


**Fig. S3.** High-dose mouse-adapted PR8 strain challenge induces a cytokine storm in the lung tissues of BALB/c mice. The relative mRNA expression levels of cytokines in mouse lung tissues were measured by qRT-PCR at 3 dpi with the PR8 strain. The test genes include pattern recognition receptor (RIG-1, TLR-2, TLR-3, TLR-7, TLR-9), type I interferon response (IFN-α, IFN-β, STAT2, IRF1, IRF3, IRF7), type II/general interferon response (IFN-γ, STAT1, IRF2), TH1/2 response (T-BET, GATA3), T/B cell (CD3, CD19), inflammatory cytokines (IL-1β, IL-6, IL-10, TNF-α and NF-κB), chemokines (CCL-2, CCL-5, CXCL-1, CXCL-10), and interferon stimulated genes (OAS, ISG-15, PKR). The fold change of mRNA expression was calculated using the 2^-ΔΔCT^ method against PBS group with GAPDH as the housekeeping gene. Each data point was assessed in triplicate. Statistical significance was determined using an unpaired two-tailed *t*-test. (* *p* < 0.05, ** *p* < 0.01, *** *p* < 0.001, **** *p* < 0.0001 and ns, not significant).


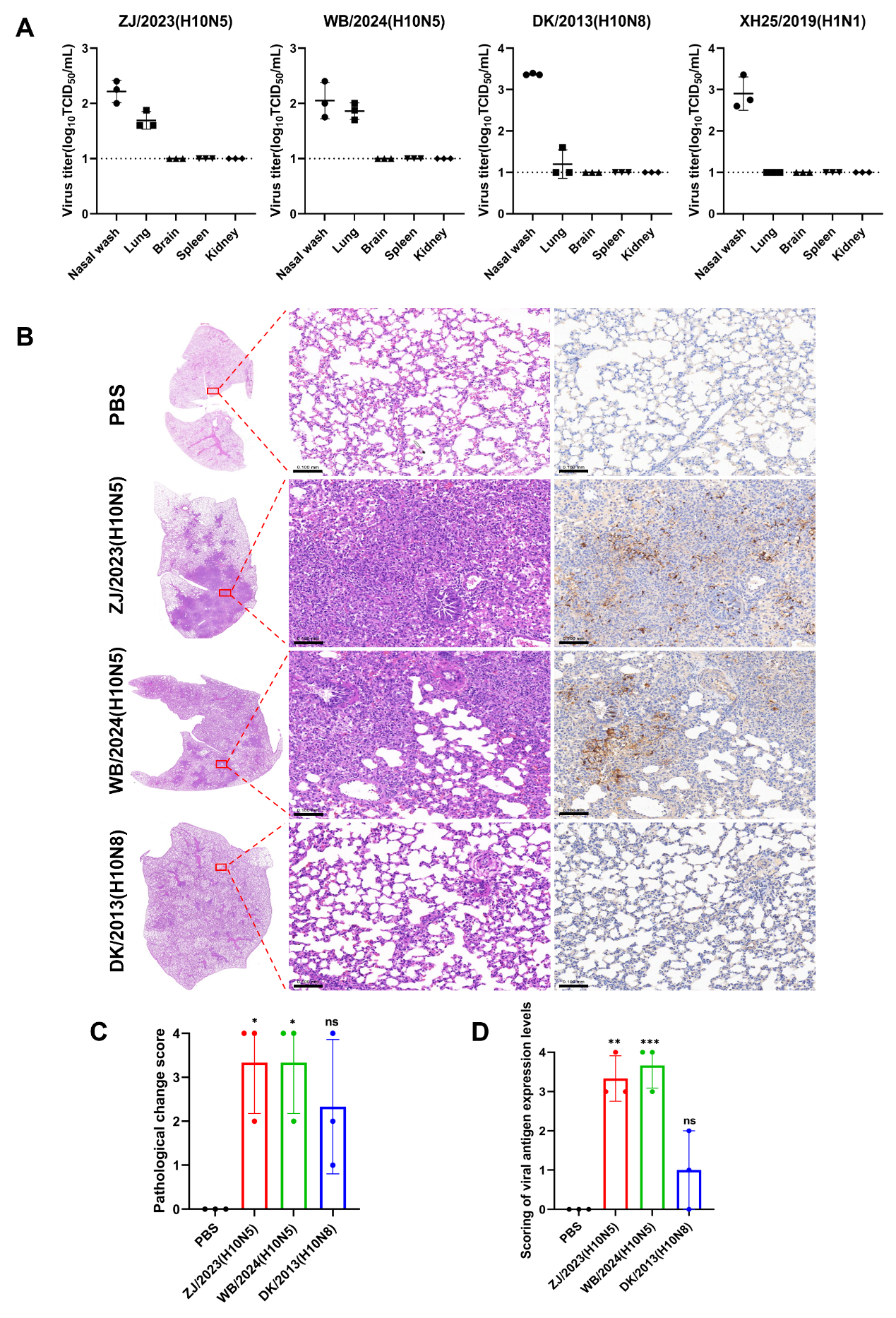


**Fig. S4.** Pathogenicity and replication of novel H10N5 viruses in guinea pigs. (A) Each group of three guinea pigs was inoculated with the indicated virus at a dose of 10^6^ TCID_50_. Euthanasia was performed three days post-infection. Nasal wash, lung, brain, spleen, and kidney tissues were collected for virus titration by TCID_50_ assay on MDCK cells. The dashed lines indicate the lower limit of detection. (B) Representative Hematoxylin and Eosin (HE) staining and immunohistochemistry (IHC) staining of the lungs of guinea pigs infected with the specified virus at 3 dpi. The standard for pathological change score and immunohistochemical score were shown in Fig. S2. All scale bars = 100 µm. Statistical significance was based on one-way ANOVA, compared with the corresponding value of PBS (* *p* < 0.05, ** *p* < 0.01, *** *p* < 0.001, **** *p* < 0.0001 and ns, not significant).


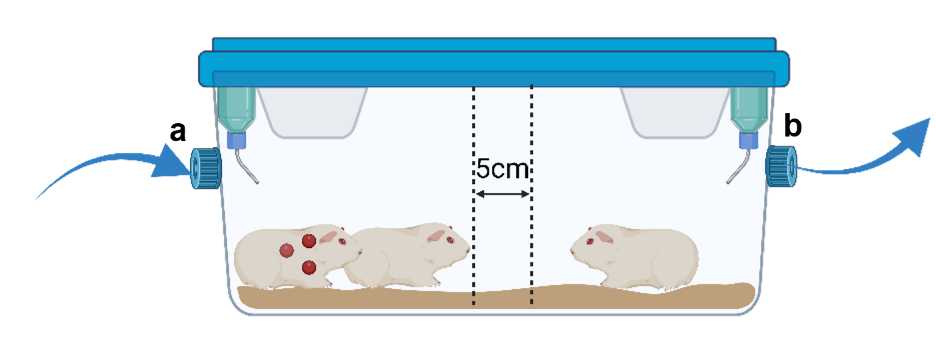


**Fig. S5.** Schematic diagram of guinea pig transmission studies. One inoculated donor guinea pig was housed on the left side of a cage. Donor guinea pig (marked with red dots) was intranasally inoculated with 10^6^ TCID_50_ viruses of ZJ/2023 (H10N5), WB/2024 (H10N5), DK/2013 (H10N8), and XH25/2019 (H1N1pdm). After 24 hours of infection, one direct-contact guinea pig was housed in the same cage as the inoculated guinea pig. At the same time, airborne-exposed guinea pig was introduced into the other side of the cage. The inoculated guinea pig and airborne-exposed guinea pig were 5 cm apart to avoid contact transmission. Negative pressure within the cage was used to direct a modest (0.3 m/s) flow of HEPA-filtered air (a) from the inoculated to the naive animals. The outlet airflow (b) was HEPA-filtered to prevent the continuous circulation of infectious influenza virus particles. There were three one-to-one transmission pairs for each virus. Image created with BioRender.com.

**Table S1.** All amino acid differences and homology between ZJ/2023 (H10N5) and WB/2024 (H10N5) virus.

| Protein | Site | ZJ/2023 (H10N5) | WB/2024 (H10N5) | Per. Ident (%) |
| --- | --- | --- | --- | --- |
| HA  (H3 numbering) | 4 | V | I | 99.11 |
|  | 128 | G | E |  |
|  | 149 | A | T |  |
|  | 158 | S | N |  |
|  | 223 | V | M |  |
| NA | 61 | T | I | 98.73 |
|  | 90 | A | V |  |
|  | 216 | R | K |  |
|  | 230 | M | I |  |
|  | 258 | I | L |  |
|  | 347 | I | V |  |
| PB2 | 82 | K | N | 99.08 |
|  | 344 | M | V |  |
|  | 356 | I | V |  |
|  | 453 | S | P |  |
|  | 482 | K | R |  |
|  | 559 | I | T |  |
|  | 724 | V | A |  |
| PB1 | 179 | M | L | 99.47 |
|  | 213 | K | N |  |
|  | 667 | V | I |  |
|  | 694 | S | N |  |
| PA | 104 | K | R | 99.58 |
|  | 337 | T | A |  |
|  | 682 | D | N |  |
| NP | 67 | A | V | 99.60 |
|  | 446 | R | K |  |
| M1 | - | - | - | 100 |
| M2 | 55 | L | F | 98.98 |
| NS1 | 33 | H | L | 98.27 |
|  | 60 | A | V |  |
|  | 139 | N | D |  |
|  | 185 | L | F |  |
| NEP | - | - | - | 100 |

**Table S2.** Key mutations in the polymerase, M1, and NS1 proteins that contribute to the increased adaptation, replication, and virulence in mammals, as well as potential critical sites responsible for aerosol transmission of the DK/2013 (H10N8).

| **Protein** | **Mutation** | **Function** | **ZJ/2023**  **(H10N5)** | **WB/2024**  **(H10N5)** | **DK/2013**  **(H10N8)** | **References** |
| --- | --- | --- | --- | --- | --- | --- |
| PB2 | L89V | Compensates for the lack of 627K | V | V | V | (1) |
|  | T271A | Enhances polymerase activity in human | T | T | T | (2) |
|  | I292V | Promotes the mammalian adaptation | I | I | V^a^ | (3) |
|  | G309D T339K R477G I495V | Compensate for the lack of 627K | D  K  G  V | D  K  G  V | D  K  G  V | (1) |
|  | I504V | Enhances polymerase activity and virulence | V | V | V | (4) |
|  | K526R |  | K | K | K | (5) |
|  | A588V | Promotes the mammalian adaptation | A | A | V^a^ | (6,7) |
|  | E627K | Induces airborne transmission | E | E | E | (1,8,9) |
|  | D701N | Enhances pathogenicity and transmission | D | D | D | (8,9) |
|  | R389K  T598M/V  L648V  T676M | Higher adaptability in mammals and promotes transmission in chicken | R  T  L  T | R  T  L  T | K^a^  V^a^  V^a^  M^a^ | (7) |
| PB1 | D3V | Increases polymerase activity and viral replication | V | V | V | (10) |
|  | L13P | Observed in human isolates | P | P | P | (11) |
|  | I368V | Mammalian adaptation | I | I | V^a^ | (12) |
|  | D622G | Increases polymerase activity and virulence | G | G | G | (13) |
| PA | A37S  I61T  V63I | Increases virulence and mammalian adaptation | A  I  V | A  I  V | S^a^  T^a^  I^a^ | (14) |
|  | A343S  K356R  S409N | Human adaptation | A  K  S | A  K  S | S^a^  R^a^  N^a^ | (15,12) |
| NP | M105V | Increases virulence in chicken | M | M | V | (16,17) |
|  | A184K | Enhances replication and virulence in chickens, enhanced IFN response | K | K | K | (18) |
| M1 | N30D | Increases virulence in mice | D | D | D | (19) |
|  | I43M | Increases virulence in mice, chickens and ducks | M | M | M | (20) |
|  | T215A | Enhances virulence in mice | A | A | A | (19) |
| NS1 | P42S | Enhances virulence in mice | S | S | S | (21) |
|  | K55E  K66E | Enhances replication in mammalian cells, decrease IF response | E  E | E  E | E  E | (22) |
|  | V149A | Increased virulence and decreased interferon response in chickens | A | A | A | (23) |

^a^: The potential mammalian adaptation amino acids sites that may facilitate the transmission of the DK/2013 (H10N8) virus between guinea pigs.

**Table S3.** Panel of BALB/c mice primers against host response genes.

| **Target** | **Forward Primer Sequence (5′-3′)** | **Reverse Primer Sequence (5′-3′)** |
| --- | --- | --- |
| GAPDH | GGTTGTCTCCTGCGACTTCA | GGTTGTCTCCTGCGACTTCA |
| RIG-I | CACTTCGTTCATCTCTGGCG | AGCCTGAATGTACTGCACCT |
| TLR2 | CTGAGAATGATGTGGGCGTG | TTAAAGGGCGGGTCAGAGTT |
| TLR3 | TTGCGTTGCGAAGTGAAGAA | TGTTCAAGAGGAGGGCGAAT |
| TLR7 | AGCAGGACCATGGAAAGTGA | TAGATTTGGCGGCATACCCT |
| TLR9 | CAAGTACACGCTCAGATGGC | CCCACTGATGCGATTGTCTG |
| IFN-α | TGACCTCAAAGCCTGTGTGA | TCCTCACTCAGTCTTGCCAG |
| IFN-β | GTCCTCAACTGCTCTCCACT | GAAGATCTCTGCTCGGACCA |
| STAT2 | GAAGATGAAGCTGCAGACGG | TTGGGCTGAGCATGTTGAAC |
| IRF1 | ACCAGAGATTGACAGCCCTC | CTCACTCAGGAGGGCAAGAA |
| IRF3 | CTGAAAACCGTGGACTTGCA | AGTCCATGTCCTCCACCAAG |
| IRF7 | TGCTGTTTGGAGACTGGCTA | CGAAATGCTTCCAGGGTACG |
| IFN-γ | CATGGCTGTTTCTGGCTGTT | TCCTTTTGCCAGTTCCTCCA |
| STAT1 | TGATTGACCTGGAGACCACC | TCAACACCTCTGAGAGCTGG |
| IRF2 | CATCGAAGGCAAGCAGTACC | GAGCTGTTGTAAGGCATCGG |
| T-BET | GTGTCTGGGAAGCTGAGAGT | GGTGAAGGACAGGAATGGGA |
| GATA3 | TCTCCAAGTGTGCGAAGAGT | TCCGGATTCAGTGGTTGGAA |
| CD3 | CAACCCAGACTATGAGCCCA | AAGAGCAAGCTGTGGAGTCT |
| CD19 | CCCAGATGGTGTCAGTCAGT | TCAACCCCATCTTCCCTTCC |
| IL-1β | ACTCATTGTGGCTGTGGAGA | TTGTTCATCTCGGAGCCTGT |
| IL-6 | TCTGCAAGAGACTTCCATCCAGTTG | AGCCTCCGACTTGTGAAGTGGT |
| IL-10 | AAGCTCCAAGACCAAGGTGT | AGCTCTGTCTAGGTCCTGGA |
| TNF-α | TGAGGTCAATCTGCCCAAGT | GGGGTCAGAGTAAAGGGGTC |
| NF-κB | CACCGGATTGAAGAGAAGCG | AGTTGAGTTTCGGGTAGGCA |
| CCL2 | CAGCTCTCTCTTCCTCCACC | TGGGATCATCTTGCTGGTGA |
| CCL5 | TGCCAACCCAGAGAAGAAGT | AGATGCCCATTTTCCCAGGA |
| CXCL1 | AGTAGAAGGGTGTTGTGCGA | CGTGCGTGTTGACCATACAA |
| CXCL10 | GTGAGAATGAGGGCCATAGG | TTTTTGGCTAAACGCTTTCAT |
| OAS | GCCGTCAATGTCGTGTGTG | ACTTGCCCTTGAGTGTGG |
| ISG-15 | TTCCTGGTGTCCGTGACTAAC | GACTGGAAAGGGTAAGACCGT |
| PKR | AGATTTCAGAGCCTGCACCT | TGGGGTATCACTGGCCATTT |

**references**

1. Li J, Ishaq M, Prudence M, Xi X, Hu T, Liu Q, Guo D. 2009. Single mutation at the amino acid position 627 of PB2 that leads to increased virulence of an H5N1 avian influenza virus during adaptation in mice can be compensated by multiple mutations at other sites of PB2. Virus Research 144:123–129.

2. Bussey KA, Bousse TL, Desmet EA, Kim B, Takimoto T. 2010. PB2 residue 271 plays a key role in enhanced polymerase activity of influenza A viruses in mammalian host cells. J Virol 84:4395–4406.

3. Gao W, Zu Z, Liu J, Song J, Wang X, Wang C, Liu L, Tong Q, Wang M, Sun H, Sun Y, Liu J, Chang K-C, Pu J. 2019. Prevailing I292V PB2 mutation in avian influenza H9N2 virus increases viral polymerase function and attenuates IFN-β induction in human cells. J Gen Virol 100:1273–1281.

4. Rolling T, Koerner I, Zimmermann P, Holz K, Haller O, Staeheli P, Kochs G. 2009. Adaptive Mutations Resulting in Enhanced Polymerase Activity Contribute to High Virulence of Influenza A Virus in Mice. Journal of Virology https://doi.org/10.1128/jvi.00212-09.

5. Song W, Wang P, Mok BW-Y, Lau S-Y, Huang X, Wu W-L, Zheng M, Wen X, Yang S, Chen Y, Li L, Yuen K-Y, Chen H. 2014. The K526R substitution in viral protein PB2 enhances the effects of E627K on influenza virus replication. Nat Commun 5:5509.

6. Xiao C, Ma W, Sun N, Huang L, Li Y, Zeng Z, Wen Y, Zhang Z, Li H, Li Q, Yu Y, Zheng Y, Liu S, Hu P, Zhang X, Ning Z, Qi W, Liao M. 2016. PB2-588 V promotes the mammalian adaptation of H10N8, H7N9 and H9N2 avian influenza viruses. Sci Rep 6:19474.

7. Li B, Su G, Xiao C, Zhang J, Li H, Sun N, Lao G, Yu Y, Ren X, Qi W, Wang X, Liao M. The PB2 co‐adaptation of H10N8 avian influenza virus increases the pathogenicity to chickens and mice https://doi.org/10.1111/tbed.14157.

8. Gao Y, Zhang Y, Shinya K, Deng G, Jiang Y, Li Z, Guan Y, Tian G, Li Y, Shi J, Liu L, Zeng X, Bu Z, Xia X, Kawaoka Y, Chen H. 2009. Identification of Amino Acids in HA and PB2 Critical for the Transmission of H5N1 Avian Influenza Viruses in a Mammalian Host. PLOS Pathogens 5:e1000709.

9. Steel J, Lowen AC, Mubareka S, Palese P. 2009. Transmission of Influenza Virus in a Mammalian Host Is Increased by PB2 Amino Acids 627K or 627E/701N. PLOS Pathogens 5:e1000252.

10. Elgendy EM, Arai Y, Kawashita N, Daidoji T, Takagi T, Ibrahim MS, Nakaya T, Watanabe Y. 2017. Identification of polymerase gene mutations that affect viral replication in H5N1 influenza viruses isolated from pigeons. Journal of General Virology 98:6–17.

11. de Jong MD, Simmons CP, Thanh TT, Hien VM, Smith GJD, Chau TNB, Hoang DM, Van Vinh Chau N, Khanh TH, Dong VC, Qui PT, Van Cam B, Ha DQ, Guan Y, Peiris JSM, Chinh NT, Hien TT, Farrar J. 2006. Fatal outcome of human influenza A (H5N1) is associated with high viral load and hypercytokinemia. Nat Med 12:1203–1207.

12. Guo Y, Ding P, Li Y, Zhang Y, Zheng Y, Yu M, Suzuki Y, Zhang H, Ping J. 2022. Genetic and biological properties of H10N3 avian influenza viruses: A potential pandemic candidate? Transbounding Emerging Dis 69.

13. Feng X, Wang Z, Shi J, Deng G, Kong H, Tao S, Li C, Liu L, Guan Y, Chen H. 2016. Glycine at Position 622 in PB1 Contributes to the Virulence of H5N1 Avian Influenza Virus in Mice. J Virol 90:1872–1879.

14. Hu M, Chu H, Zhang K, Singh K, Li C, Yuan S, Chow BKC, Song W, Zhou J, Zheng B-J. 2016. Amino acid substitutions V63I or A37S/I61T/V63I/V100A in the PA N-terminal domain increase the virulence of H7N7 influenza A virus. Sci Rep 6:37800.

15. Xu G, Zhang X, Gao W, Wang C, Wang J, Sun H, Sun Y, Guo L, Zhang R, Chang K-C, Liu J, Pu J. 2016. Prevailing PA Mutation K356R in Avian Influenza H9N2 Virus Increases Mammalian Replication and Pathogenicity. Journal of Virology 90:8105–8114.

16. Tada T, Suzuki K, Sakurai Y, Kubo M, Okada H, Itoh T, Tsukamoto K. 2011. Emergence of Avian Influenza Viruses with Enhanced Transcription Activity by a Single Amino Acid Substitution in the Nucleoprotein during Replication in Chicken Brains. Journal of Virology 85:10354–10363.

17. Tada T, Suzuki K, Sakurai Y, Kubo M, Okada H, Itoh T, Tsukamoto K. 2011. NP body domain and PB2 contribute to increased virulence of H5N1 highly pathogenic avian influenza viruses in chickens. J Virol 85:1834–1846.

18. Wasilenko JL, Sarmento L, Pantin-Jackwood MJ. 2009. A single substitution in amino acid 184 of the NP protein alters the replication and pathogenicity of H5N1 avian influenza viruses in chickens. Arch Virol 154:969–979.

19. Fan S, Deng G, Song J, Tian G, Suo Y, Jiang Y, Guan Y, Bu Z, Kawaoka Y, Chen H. 2009. Two amino acid residues in the matrix protein M1 contribute to the virulence difference of H5N1 avian influenza viruses in mice. Virology 384:28–32.

20. Nao N, Kajihara M, Manzoor R, Maruyama J, Yoshida R, Muramatsu M, Miyamoto H, Igarashi M, Eguchi N, Sato M, Kondoh T, Okamatsu M, Sakoda Y, Kida H, Takada A. 2015. A Single Amino Acid in the M1 Protein Responsible for the Different Pathogenic Potentials of H5N1 Highly Pathogenic Avian Influenza Virus Strains. PLoS One 10:e0137989.

21. Jiao P, Tian G, Li Y, Deng G, Jiang Y, Liu C, Liu W, Bu Z, Kawaoka Y, Chen H. 2008. A single-amino-acid substitution in the NS1 protein changes the pathogenicity of H5N1 avian influenza viruses in mice. J Virol 82:1146–1154.

22. Li J, Zhang K, Chen Q, Zhang X, Sun Y, Bi Y, Zhang S, Gu J, Li J, Liu D, Liu W, Zhou J. 2018. Three amino acid substitutions in the NS1 protein change the virus replication of H5N1 influenza virus in human cells. Virology 519:64–73.

23. Li Z, Jiang Y, Jiao P, Wang A, Zhao F, Tian G, Wang X, Yu K, Bu Z, Chen H. 2006. The NS1 Gene Contributes to the Virulence of H5N1 Avian Influenza Viruses. J Virol 80:11115–11123.
